# Design and green synthesis of novel quinolinone derivatives of potential anti-breast cancer activity against MCF-7 cell line targeting multi-receptor tyrosine kinases

**DOI:** 10.1080/14756366.2021.1944126

**Published:** 2021-07-01

**Authors:** Mohamed Mokhtar, Khadijah S. Alghamdi, Nesreen S. Ahmed, Dina Bakhotmah, Tamer S. Saleh

**Affiliations:** a Chemistry Department, Faculty of Science, King Abdulaziz University, Jeddah, Saudi Arabia; b Chemistry Department, Faculty of Science, Albaha University, Albaha, Saudi Arabia; c Department of Therapeutic Chemistry, National Research Centre, Cairo,Egypt; d Department of Chemistry, University of Jeddah, College of Science, Jeddah, Saudi Arabia; e Green Chemistry Department, National Research Centre, Giza, Egypt

**Keywords:** 4,6,7,8-Tetrahydroquinolin-5(1H)-one, HER-2 inhibitors, breast cancer, apoptosis, molecular docking study

## Abstract

A new set of 4,6,7,8-tetrahydroquinolin-5(1H)-ones were designed as cytotoxic agents against breast cancer cell line (MCF-7) and synthesised under ultrasonic irradiation using chitosan decorated copper nanoparticles (CS/CuNPs) catalyst. The new compounds **4b**, **4j**, **4k**, and **4e** exhibited the most potent cytotoxic activity of IC_50_ values (0.002 − 0.004 µM) comparing to Staurosporine of IC_50_; 0.005 μM. The latter derivatives exhibited a promising safety profile against the normal human WI38 cells of IC_50_ range 0.0149 − 0.048 µM. Furthermore, the most promising cytotoxic compounds **4b**, **4j** were evaluated as multi-targeting agents against the RTK protein kinases; EGFR, HER-2, PDGFR-β, and VEGFR-2. Compound 4j showed promising inhibitory activity against HER-2 and PDGFR-β of IC_50_ values 0.17 × 10^−3^, 0.07 × 10^−3^ µM in comparison with the reference drug sorafenib of IC_50_; 0.28 × 10^−3^, 0.13 × 10^−3^ µM, respectively. In addition, **4j** induced apoptotic effect and cell cycle arrest at G2/M phase preventing the mitotic cycle in MCF-7 cells.

## Graphical Abstract

**Figure:**
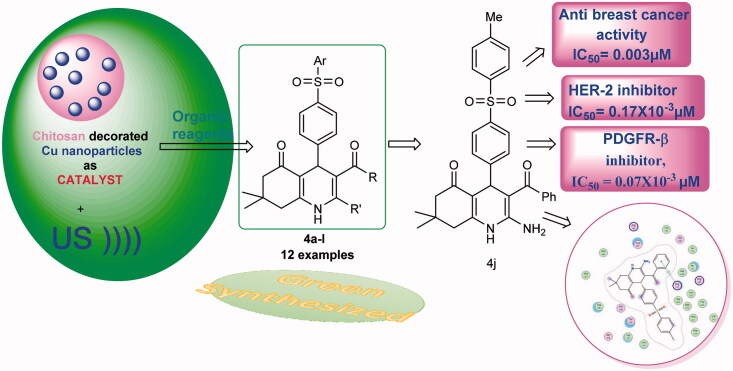

## 1. Introduction

Breast cancer is one of the most common causes that threatens women’s health impacting ∼15% of all cancer deaths occurred in women all over the world^1^^,^^2^. The incidence of breast cancer is increasing in all the regions of globe. Although chemo and radiotherapies are considered the most important prime option for the treatment of cancer disease,^3^ the drug-induced toxicity to the normal cells and drug-resistance constitute problems that still need to be resolved^4^. Therefore, the discovery of new selective and safer anticancer agents is still a great interest in the field of medicinal chemistry^5^.

**Figure 1.**
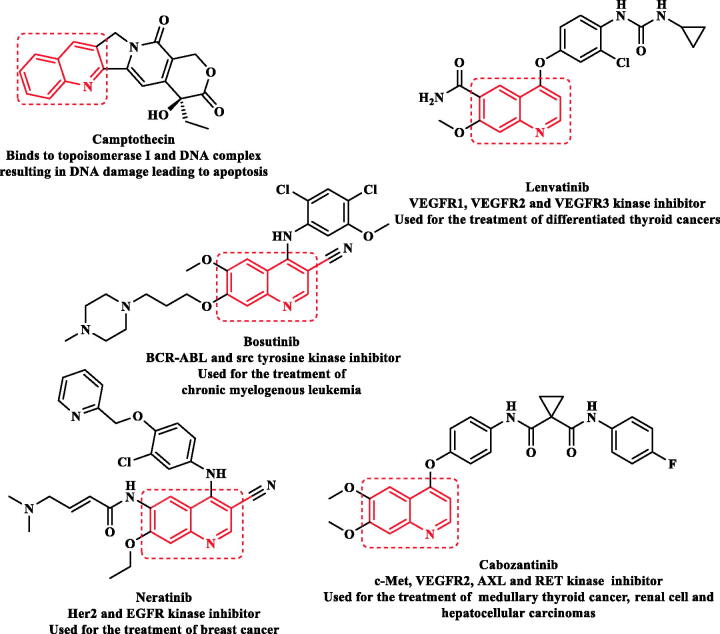
Quinoline-based multi-kinase inhibitors approved by FDA.

Receptor tyrosine kinases (RTKs) are transmembrane proteins. They are composed of many triggered domains when a ligand binds to their extracellular regions, activating signalling cascades downstream^6^^,^^7^. RTKs such as; epidermal growth factor receptor (EGFR), vascular endothelial growth factor receptor-2 (VEGFR-2), and PDGFR play crucial roles in controlling multiple cellular processes, including cell proliferation, differentiation, survival, and apoptosis. Mutations or deletions in gene functions can result in uncontrolled expression of protein kinases, which can lead to tumour development, angiogenesis, and metastasis. Thus, RTKs are considered novel drug targets to develop tyrosine kinase inhibitors^6–9^. In addition, these suppressors can inhibit the overexpression of tyrosine kinase and maintain its physiological balance^10^. The US FDA approves more than 60 small-molecule protein kinase inhibitors. Most of them are multi-targeting suppressors synergistically functioning on many signalling pathways by interacting with each target simultaneously^11^. Targeting multiple kinases increases the anticancer potency as well as reduces the risk of developing drug resistance^11–13^ (Figure 1).

Quinoline is naturally present in many alkaloids having potent antitumor activity, for example, camptothecin^14^ (Figure 1). Quinoline scaffold and its related derivatives represent a broad spectrum of pharmacological activities, especially in drug discovery of new anticancer agents^15–18^. Food and Drug Administration (FDA) has approved various quinoline small molecules acting as protein kinases inhibitors for clinical uses in cancer disease^19–22^ (Figure 1). The occurrence of the nitrogen atom in the quinoline nucleus withdraws electrons by resonance and interferes with the equal distribution of π electron density. It has been reported that the quinoline nucleus has a great tendency to bind to the active site of various proteins *via* the formation of hydrogen bonds with its nitrogen atom and π–π stacking complexes with complementary amino acid residues^15^^,^^16^.

**Figure 2.**
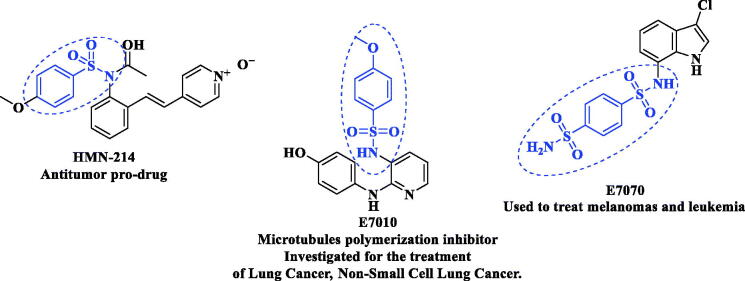
Various sulphonamide candidates of antitumor activity *via* different various modes of actions.

Furthermore, multiple sulphonyl compounds have been reported to inhibit the growth of various human tumour cell lines. Various sulphonamide derivatives, such as HMN-214, E7010 (ABT-751), and E7070 (Indisulam), represented antitumor activity through different various modes of actions as multidrug resistance down-regulation, inhibition of tubulin polymerisation as well as RTKs inhibition^23–27^ (Figure 2).

Molecular hybridisation currently appears as a promising drug design strategy, specifically in discovering new anticancer drugs^28^^,^^29^. It has been reported that conjugating of two or more pharmacophores in the same molecular architecture might decrease the risk of drug–drug interactions, overcome the problem of drug resistance as well as enhance the biological efficacy *via* the binding with different targets as one single entity^30^.

**Figure 3.**
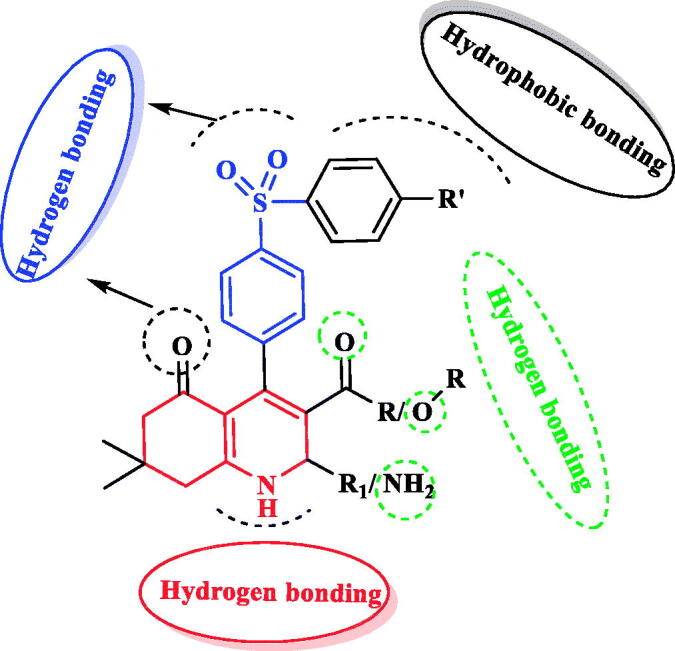
The proposed hypothetic model for the new tetrahydroquinoline – phenylsulfonyl derivatives.

Based on the above findings, in continuation of our recent work of using green synthetic approaches to develop a variety of heterocyclic systems with great biological importance^31–34^, and in an attempt to prepare new potent anti-breast cancer leads of potential suppression activity against different RTKs EGFR, HER-2, PDGFR-β, and VEGFR-2, this study deals with the synthesis of new tetrahydroquinolines hybridised with other substituted phenylsulfonyl-phenyl moieties at C-4 position and conjugated with different groups at C-2 and C-3 positions (Figure 3). It has been taken in consideration the effect of molecular orientation, ring size variation and the occurrence of different heteroatoms that could provide hydrogen binding with various RTKs binding pockets (Figure 3). The cytotoxic activity against human breast cancer cells (MCF-7) was evaluated for all the new prepared analogues. In addition, *in vitro* multi-targeting inhibition assessment against EGFR, HER-2, PDGFR-β, and VEGFR-2 of the most active cytotoxic candidates was also carried out. Extra investigations of different mechanistic pathways such as cell cycle analysis and apoptosis were evaluated for the most promising compound as a representative example for the new active analogues. Furthermore, molecular modelling studies were performed to explore the modes of interaction between the promising target compounds and the vital amino acids residues of different kinases to ascertain binding stability and the relationship between their physicochemical characteristics and their favourable suppression effects.

## 2. Materials and methods

### 2.1. Chemistry

All organic solvents were purchased from commercial sources and used as received unless otherwise stated. All other chemicals were purchased from Merck, Aldrich, or Acros and used without further purification. Thin-layer chromatography (TLC) was performed on precoated Merck 60 GF254 silica gel plates with a fluorescent indicator, and detection by means of UV light at 254 and 360 nm. The melting points were measured on a Stuart melting point apparatus and are uncorrected. IR spectra were recorded on a Smart iTR, which is an ultrahigh-performance, versatile Attenuated Total Reflectance (ATR) sampling accessory on the Nicolet iS10 FT-IR spectrometer. The NMR spectra were recorded on a Bruker Avance III 400 (9.4 T, 400.13 MHz for ^1^H, 100.62 MHz for ^13 ^C) spectrometer with a 5-mm BBFO probe, at 298 K and a Bruker High Performance Digital FT-NMR Spectrometer Avance III 850 MHz. Chemical shifts (δ in ppm) are given relative to internal solvent, DMSO-d_6_ 2.50 for ^1^H and 39.50 for ^13 ^C, CDCl_3_ 7.25 for ^1^H and 77.7 was used as an external standard. Mass spectra were recorded on a Thermo ISQ Single Quadrupole GC-MS. Elemental analyses were carried out on a Euro Vector instrument C, H, N, S analyser EA3000 Series. Sonication was performed by Techno-gaz sonicator (with a frequency of 37 kHz and ultrasonic peak max. 320 W).

The catalyst (CS/CuNPs)^35^, 4-(phenylsulfonyl)benzaldehyde (**2a**)^36^ and 4-tosylbenzaldehyde (**2b**)^37^ were prepared according to reported literature.

### 2.2. General methods for the synthesis of 4,6,7,8-tetrahydroquinolin-5(1H)-one derivatives (4a–l)

#### 2.2.1. Silent reactions

A mixture of dimedone **(1)** (1 mmol), different aldehydes **2a, b** (1 mmol), active methylene compounds **3a–f** (1 mmol) and ammonium acetate (9 mmol) in ethanol (25 ml) containing a catalytic amount of Cu-chitosan NPs (0.1 g) was refluxed at 60 °C for the appropriate time (*cf.* Table 1) until completion of the reaction (monitored by TLC). The reaction mixture was filtered to separate the catalyst; then, the filtrate was cooled at room temperature, and the reliable product obtained was filtered, dried, and purified by recrystallisation from ethanol.

**Table 1.** Synthesis of new 4,6,7,8-tetrahydroquinolin-5(1H)-ones scaffold **4a–l** under both ultrasound and conventional conditions.

Compound	Conventional conditions		Ultrasound conditions	
	Time (h)	Yield %	Time (min)	Yield%
	5	80	20	93
	5	78	20	92
	6	84	25	95
	6	80	30	92
	6	78	30	90
	6	78	30	90
	5	84	25	95
	5	82	25	95
	5	84	25	93
	6	79	30	92
	6	78	30	92
	6	78	30	90

#### 2.2.2. Sonicated reactions

These processes were performed on the same scale described above for silent reactions. All The reactions were kept at 60 °C, which was attained by adding or removing water in an ultrasonic bath (the temperature inside the reaction vessel was 58–63 °C). The sonochemical reactions were continued for a suitable time (*cf.* Table 1) until the starting materials were no longer detectable by TLC. Then, the catalyst was separated, and the products obtained were purified as described above in silent reaction procedures. The synthesised compounds with their physical data are listed below.

##### 2.2.2.1. Methyl 2,7,7-trimethyl-5-oxo-4–(4-(phenylsulfonyl)phenyl)-1,4,5,6,7,8-hexahydroquinoline-3-carboxylate (4a)

Mp 260–262 °C. IR (KBr) *v*_max_/cm^−1^: 3213 (NH), 1714, 1688 (2 C = O), 1310, 1211 (SO_2_); ^1^H NMR (400 MHz, DMSO-d_6_): δ 0.80 (s, 3H, CH_3_), 0.98 (s, 3H, CH_3_), 1.97 (d, 1H, *J* = 16 Hz, H-8a), 2.15 (d, 1H, *J* = 16 Hz, H-8b), 2.28 (s, 3H, CH_3_), 2.33 (d, 1H, *J* = 16.4 Hz, H-6a), 2.41 (d, 1H, *J* = 16.4 Hz, H-6b), 3.49 (s, 3H, CH_3_ ester), 4.91 (s, 1H, CH-4), 7.35 (d, 2H, *J* = 7.6 Hz, ArH), 7.39 (d, 2H, *J =* 7.72 Hz, ArH), 7.76–7.80 (m, 5H, ArH), 9.21 (br’s, 1H, NH, D_2_O exchangeable). ^13 ^C NMR (100 MHz, DMSO-d_6_): δ 18.8, 27.1, 29.3, 32.7, 36.7, 50.5, 51.3, 102.7, 109.6, 127.5, 127.8, 132.1, 138.9, 144.7, 153.4, 167.4, 194.8; MS *m/z* (%): 465 (M^+^, 40.9). Anal. for C_26_H_27_NO_5_S: C, 67.08; H, 5.85; N, 3.01; S, 6.89. found: C, 67.29; H, 5.79; N, 2.90; S, 6.83%.

##### 2.2.2.2. Ethyl 7,7-dimethyl-5-oxo-4–(4-(phenylsulfonyl)phenyl)-2-(trifluoromethyl)-1,4,5,6,7,8-hexahydroquinoline-3-carboxylate (4b)

Mp 245–247 °C. IR (KBr) *v*_max_/cm^−1^: 3221 (NH), 1717, 1688 (2 C = O), 1314, 1210 (SO_2_); ^1^H NMR (400 MHz, DMSO-d_6_): δ 0.85 (s, 3H, CH_3_), 0.98 (s, 3H, CH_3_), 1.13 (t, 3H, *J =* 7 Hz CH_3_ ester) 1.96 (d, 1H, *J* = 16 Hz, H-8a), 2.16 (d, 1H, *J* = 16 Hz, H-8b), 2.35 (d, 1H, *J* = 16.4 Hz, H-6a), 2.43 (d, 1H, *J* = 16.4 Hz, H-6a), 4.19 (q, 2H, *J =* 7 Hz, CH_2_ Ester), 4.92 (s, 1H, CH-4), 7.35 (d, 2H, *J* = 7.6 Hz, ArH), 7.39 (d, 2H, *J =* 7.72 Hz, ArH), 7.71–7.86 (m, 5H, ArH), 9.22 (br’s, 1H, NH, D_2_O exchangeable). ^13 ^C NMR (100 MHz, DMSO-d_6_): δ 18.9, 27.1, 29.3, 32.7, 36.7, 51.3, 62.0, 102.6, 109.5, 123.2, 127.6, 127.7, 130.2, 134.9, 138.9, 141.7, 146.7, 150.6, 153.6, 167.4, 194.7; MS *m/z* (%): 533 (M^+^, 51.5). Anal. for C_27_H_26_F_3_NO_5_S: C, 60.78; H, 4.91; N, 2.63; S, 6.01. found: C, 60.99; H, 4.87; N, 2.53; S, 5.94%.

##### 2.2.2.3. Ethyl 2-amino-7,7-dimethyl-5-oxo-4–(4-(phenylsulfonyl)phenyl)-1,4,5,6,7,8-hexahydroquinoline-3-carboxylate (4c)

Mp 238–240 °C. IR (KBr) *v*_max_/cm^−1^: 3389, 3282 (NH_2_), 3216 (NH), 1712, 1689 (2 C = O), 1310, 1196 (SO_2_); ^1^H NMR (400 MHz, CDCl_3_): δ 0.96 (s, 3H, CH_3_), 1.09 (s, 3H, CH_3_), 1.14(t, 3H, *J =* 7.2 Hz, CH_3_ ester), 1.84 (br’s, 2H, NH_2_, D_2_O exchangeable), 2.17 (dd, 2H, *J* = 14 Hz, H-8a and H8b), 2.41 (s, 2H, CH_2_-6), 4.03(q, 2H, *J* = 7.2 Hz, CH_2_ ester), 4.66 (s, 1H, C4 –H), 7.15 (d, 2H, *J* = 7.6 Hz, ArH), 7.21 (d, 2H, *J =* 7.72 Hz, ArH), 7.39–7.78 (m, 5H, ArH), 9.54 (br’s, 1H, NH, D_2_O exchangeable). ^13 ^C NMR (100 MHz, CDCl_3_): δ 14.2, 27.4, 29.1, 32.2, 33.5, 40.7, 50.7, 59.8, 80.3, 116.4, 122.0, 126.9, 127.9, 129.6, 131.6, 136.0, 144.5, 149.5, 158.4, 161.5, 168.9, 196.4; MS *m/z* (%): 480 (M^+^, 28.7). Anal. for C_26_H_28_N_2_O_5_S: C, 64.98; H, 5.87; N, 5.83; S, 6.67. found: C, 65.20; H, 5.81; N, 5.75; S, 6.59%.

##### 2.2.2.4. 2-Amino-3-benzoyl-7,7-dimethyl-4–(4-(phenylsulfonyl)phenyl)-4,6,7,8-tetrahydroquinolin-5(1H)-one (4d)

Mp > 300 °C. IR (KBr) *v*_max_/cm^−1^: 3339, 3291 (NH_2_), 3222 (NH), 1699, 1685 (2 C = O), 1310, 1206 (SO_2_); ^1^H NMR (850 MHz, CDCl_3_): δ 0.96 (s, 3H, CH_3_), 1.09 (s, 3H, CH_3_), 2.18 (dd, 2H, *J* = 14 Hz, H-8a and H-8b), 2.42 (dd, 2H, *J* = 16.9 Hz, H-6a and H-6b), 4.65 (s, 1H, CH-4), 6.21 (br’s, 2H, NH_2_, D_2_O exchangeable), 6.82 (d, 2H, *J* = 7.4 Hz, ArH), 7.14 (d, 2H, *J =* 7.6 Hz, ArH), 7.33 (d, 2H, *J =* 7.1 Hz, ArH), 7.56–7.81 (m, 4H, ArH), 8.11–8.48 (m, 4 h, ArH), 9.31 (br’s, 1H, NH, D_2_O exchangeable). ^13 ^C NMR (213 MHz, CDCl_3_): δ 27.3, 29.1, 32.3, 33.4, 40.6, 50.7, 80.2, 50.5, 51.3, 116.3, 126.2, 127.4, 128.1, 129.7, 129.9, 130.1, 130.8, 134.2, 137.3, 144.9, 145.1, 158.3, 168.9, 190.7, 196.5; MS *m/z* (%): 512 (M^+^, 39.1). Anal. for C_30_H_28_N_2_O_4_S: C, 70.29; H, 5.51; N, 5.46; S, 6.25. found: C, 70.52; H, 5.47; N, 5.36; S, 6.17%.

##### 2.2.2.5. 2-Amino-7,7-dimethyl-3–(4-methylbenzoyl)-4–(4-(phenylsulfonyl)phenyl)-4,6,7,8-tetrahydroquinolin-5(1H)-one (4e)

Mp > 300 °C. IR (KBr) *v*_max_/cm^−1^: 3331, 3291 (NH_2_), 3226 (NH), 1691, 1683 (2 C = O), 1314, 1201(SO_2_); ^1^H NMR (850 MHz, CDCl_3_): δ 0.96 (s, 3H, CH_3_), 1.09 (s, 3H, CH_3_), 2.17 (dd, 2H, *J* = 16.15 Hz, H-8a and H-8b), 2.41 (dd, 2H, *J* = 17.83 Hz, H-6a and H-6b), 2.58 (s, 3H, CH_3_), 4.69 (s, 1H, C4 –H), 6.26 (br’s, 2H, NH_2_, D_2_O exchangeable), 6.87 (d, 2H, *J =* 8.5 Hz), 6.88 − 6.89 (m, 5H, ArH), 7.20–7.23 (m, 4H, Ar H), 7.27 (d, 2H, *J* = 8.4 Hz, ArH), 9.05 (br’s, 1H, NH, D_2_O exchangeable). ^13 ^C NMR (213 MHz, CDCl_3_): δ 18.2, 27.3, 29.1, 32.2, 33.2, 40.6, 50.7, 80.5, 116.6, 128.8, 129.6, 129.9, 131.2, 133.6, 137.4, 139.2, 141.6, 158.3, 169.0, 189.9, 196.5; MS *m/z* (%): 526 (M^+^, 74.3). Anal. for C_31_H_30_N_2_O_4_S: C, 70.70; H, 5.74; N, 5.32; S, 6.09. found: C, 70.88; H, 5.71; N, 5.24; S, 6.04%.

##### 2.2.2.6. 2-Amino-3–(4-bromobenzoyl)-7,7-dimethyl-4–(4-(phenylsulfonyl)phenyl)-4,6,7,8-tetrahydroquinolin-5(1H)-one (4f)

Mp > 300 °C. IR (KBr) *v*_max_/cm^−1^: 3310, 3296 (NH_2_), 3221 (NH), 1691, 1682 (2 C = O), 1305, 1193 (SO_2_); ^1^H NMR (850 MHz, DMSO-d_6_): δ 0.96 (s, 3H, CH_3_), 1.04 (s, 3H, CH_3_), 2.13 (dd, 2H, *J* = 16.00 Hz, H-8a and H-8b), 2.42 (dd, 2H, *J* = 16.4 Hz, H-6a and H-6b), 4.62 (s, 1H, C4 -H), 6.23 (br’s, 2H, NH_2_, D_2_O exchangeable), 6.91 − 7.16 (m, 7H, ArH), 7.33–7.52 (m, 4H, Ar H), 7.65 (d, 2H, *J* = 8.2 Hz, ArH), 9.11 (br’s, 1H, NH, D_2_O exchangeable). ^13 ^C NMR (213 MHz, CDCl_3_): δ 27.3, 29.1, 32.2, 33.2, 40.6, 50.7, 80.5, 116.6, 128.8, 129.6, 129.9, 131.2, 133.6, 137.4, 139.2, 141.6, 158.3, 169.0, 189.9, 196.5; MS *m/z* (%): 591 (M^+^, 77.1), 593 (M^+^+2, 76.8). Anal. for C_30_H_27_BrN_2_O_4_S: C, 60.92; H, 4.60; N, 4.74; S, 5.42. found: C, 61.11; H, 4.55; N, 4.65; S, 5.37%.

##### 2.2.2.7. Methyl 2,7,7-trimethyl-5-oxo-4–(4-tosylphenyl)-1,4,5,6,7,8-hexahydroquinoline-3-carboxylate (4g)

Mp 236–238 °C. IR (KBr) *v*_max_/cm^−1^: 3218 (NH), 1723, 1674 (2 C = O), 1312, 1206 (SO_2_); ^1^H NMR (850 MHz, CDCl_3_): δ 0.91 (s, 3H, CH_3_), 1.01 (s, 3H, CH_3_), 2.02 (d, 1H, *J* = 16 Hz, H-8a), 2.09 (d, 1H, *J* = 16 Hz, H-8b), 2.33 (s, 3H, CH_3_), 2.34 (s, 3H, CH_3_), 2.36 (s, 2H, CH_2_-6), 4.42 (s, 1H, C4 -H), 3.16 (s, 3H, CH_3_ ester), 4.41 (s, 1H, CH-4), 6.70 (d, 2H, *J* = 7.6 Hz, ArH), 6.87 (d, 2H, *J =* 7.8 Hz, ArH), 7.56–7.81 (m, 4H, ArH), 9.12 (br’s, 1H, NH, D_2_O exchangeable). ^13 ^C NMR (213 MHz, CDCl_3_): δ 19.2, 21.3, 27.3, 28.3, 32.2, 39.5, 40.8, 49.7, 50.2, 104.2, 117.6, 128.8, 129.4, 134.1, 136.1, 153.6, 154.2, 156.1, 164.3, 197.6; MS *m/z* (%): 479 (M^+^, 49), Anal. for C_27_H_29_NO_5_S: C, 67.62; H, 6.10; N, 2.92; S, 6.68. found: C, 67.80; H, 6.06; N, 2.83; S, 6.63%.

##### 2.2.2.8. Ethyl 7,7-dimethyl-5-oxo-4–(4-tosylphenyl)-2-(trifluoromethyl)-1,4,5,6,7,8-hexahydroquinoline-3-carboxylate (4h)

Mp 241–243 °C. IR (KBr) *v*_max_/cm^−1^: 3231(NH), 1726, 1684 (2 C = O), 1303, 1215 (SO_2_); ^1^H NMR (400 MHz, DMSO-d_6_): δ 0.89 (s, 3H, CH_3_), 1.01 (s, 3H, CH_3_), 1.16 (t, 3H, *J =* 7 Hz CH_3_ ester), 2.01 (d, 1H, *J* = 16 Hz, H-8a), 2.14 (d, 1H, *J* = 16 Hz, H-8b), 2.33 (d, 1H, *J* = 16.4 Hz, H-6a), 2.43 (d, 1H, *J* = 16.4 Hz, H-6b), 2.48 (s, 3H, CH_3_), 4.12 (q, 2H, *J =* 7 Hz, CH_2_ Ester), 4.91 (s, 1H, CH-4), 7.31 (d, 2H, *J* = 7.00 Hz, ArH), 7.41–7.56 (m, 4H, ArH), 7.58 (d, 2H, *J =* 7.72 Hz, ArH), 9.26 (br’s, 1H, NH, D_2_O exchangeable). ^13 ^C NMR (100 MHz, DMSO-d_6_): δ 14.3, 18.2, 27.3, 29.1, 32.5, 37.2, 43.6, 51.1, 62.0, 100.4, 113.5, 124.2, 128.6, 129.2, 129.8, 130.2, 136.7, 138.2, 141.8, 146.7, 150.6, 153.6, 168.6, 194.8; MS *m/z* (%): 547 (M^+^, 39), Anal. for C_28_H_28_F_3_NO_5_S: C, 61.42; H, 5.15; N, 2.56; S, 5.85. found: C, 61.63; H, 5.09; N, 2.46; S, 5.79%.

##### 2.2.2.9. Ethyl 2-amino-7,7-dimethyl-5-oxo-4–(4-tosylphenyl)-1,4,5,6,7,8-hexahydroquinoline-3-carboxylate (4i)

Mp 281–283 °C. IR (KBr) *v*_max_/cm^−1^: 3394, 3286 (NH_2_), 3231(NH), 1726, 1684 (2 C = O), 1303, 1215 (SO_2_); ^1^H NMR (400 MHz, DMSO-d_6_): δ 0.92 (s, 3H, CH_3_), 0.99 (s, 3H, CH_3_), 1.12(t, 3H, *J =* 7.2 Hz, CH_3_ ester), 1.89 (br’s, 2H, NH_2_, D_2_O exchangeable), 2.11 (dd, 2H, *J* = 14 Hz, H-8a and H8b), 2.36 (s, 2H, CH_2_-6), 2.44 (s, 3H, CH_3_), 4.11(q, 2H, *J* = 7.2 Hz, CH_2_ ester), 4.71 (s, 1H, C4 –H), 7.15 (d, 2H, *J* = 7.2 Hz, ArH), 7.21 (d, 2H, *J =* 7.6 Hz, ArH), 7.36–7.52 (m, 5H, ArH), 9.21 (br’s, 1H, NH, D_2_O exchangeable). ^13 ^C NMR (100 MHz, CDCl_3_): δ 14.3, 18.9, 27.3, 29.6, 32.4, 35.2, 41.7, 52.4, 59.6, 83.1, 117.7, 127.6, 127.7, 129.7, 130.6, 133.9, 136.3, 138.3, 145.5, 149.5, 161.9, 168.7, 194.5; .0MS *m/z* (%): 494 (M^+^, 62.9), Anal. for C_27_H_30_N_2_O_5_S: C, 65.57; H, 6.11; N, 5.66; S, 6.48. found: C, 65.74; H, 6.06; N, 2.39; S, 6.41%.

##### 2.2.2.10. 2-Amino-3-benzoyl-7,7-dimethyl-4–(4-tosylphenyl)-4,6,7,8-tetrahydroquinolin-5(1H)-one (4j)

Mp > 300 °C. IR (KBr) *v*_max_/cm^−1^: 3382, 3267(NH_2_), 3211(NH), 1691, 1681 (2 C = O), 1310, 1211 (SO_2_); ^1^H NMR (850 MHz, CDCl_3_): δ 0.94 (s, 3H, CH_3_), 1.01 (s, 3H, CH_3_), 2.22 (dd, 2H, *J* = 14 Hz, H-8a and H-8b), 2.31 (dd, 2H, *J* = 16.9 Hz, H-6a and H-6b), 2.38 (s, 3H, CH_3_), 4.52 (s, 1H, CH-4), 6.28 (br’s, 2H, NH_2_, D_2_O exchangeable), 6.79 (d, 2H, *J* = 7.8 Hz, ArH), 7.32 (d, 2H, *J =* 7.4 Hz, ArH), 7.46–7.71 (m, 4H, ArH), 7.77–8.01 (m, 4H, ArH), 9.31 (br’s, 1H, NH, D_2_O exchangeable). ^13 ^C NMR (213 MHz, CDCl_3_): δ 18.23, 27.3, 29.2, 32.4, 34.2, 40.8, 50.1, 81.3, 50.5, 51.3, 116.3, 126.2, 127.4, 128.1, 129.7, 129.9, 130.1, 130.8, 134.2, 137.3, 144.9, 145.1, 158.3, 168.9, 190.7, 196.5; MS *m/z* (%): 526 (M^+^, 41.3), Anal. for C_31_H_30_N_2_O_4_S: C, 70.70; H, 5.74; N, 5.32; S, 6.09. found: C, 70.87; H, 5.69; N, 5.25; S, 6.02%.

##### 2.2.2.11. 2-Amino-7,7-dimethyl-3–(4-methylbenzoyl)-4–(4-tosylphenyl)-4,6,7,8-tetrahydroquinolin-5(1H)-one (4k)

Mp > 300 °C. IR (KBr) *v*_max_/cm^−1^: 3379, 3229(NH_2_), 3211(NH), 1690, 1676 (2 C = O), 1310, 1196 (SO_2_); ^1^H NMR (850 MHz, CDCl_3_): δ 0.93 (s, 3H, CH_3_), 0.97 (s, 3H, CH_3_), 2.01 (dd, 2H, *J* = 16 Hz, H-8a and H-8b), 2.22 (dd, 2H, *J* = 16.2 Hz, H-6a and H-6b), 2.36 (s, 3H, CH_3_), 2.39 (s, 3H, CH_3_), 4.61 (s, 1H, C4 –H), 6.01 (br’s, 2H, NH_2_, D_2_O exchangeable), 7.22–7.41 (m, 4H, ArH), 7.49(d, 2H, *J* = 7.2 Hz, ArH), 7.56–7.67 (m, 4H, ArH), 7.77 (d, 2H, *J =* 7.8 Hz, ArH), 9.23 (br’s, 1H, NH, D_2_O exchangeable). ^13 ^C NMR (213 MHz, CDCl_3_): δ 18.7, 19.1, 27.2, 29.4, 32.8, 37.1, 39.9, 51.1, 80.3, 116.5, 128.5, 128.8, 129.5, 129.7, 130.3, 130.9, 132.1, 138.3, 138.9, 139.0, 144.1, 152.5, 156.1, 168.9, 189.2, 195.6; MS *m/z* (%): 540 (M^+^, 65.1), Anal. for C_32_H_32_N_2_O_4_S: C, 71.09; H, 5.97; N, 5.18; S, 5.93. found: C, 71.29; H, 5.93; N, 5.08; S, 5.87%.

##### 2.2.2.12. 2-Amino-3–(4-bromobenzoyl)-7,7-dimethyl-4–(4-tosylphenyl)-4,6,7,8-tetrahydroquinolin-5(1H)-one (4l)

Mp > 300 °C. IR (KBr) *v*_max_/cm^−1^: 3379, 3229(NH_2_), 3211(NH), 1690, 1676 (2 C = O), 1310, 1196 (SO_2_); ^1^H NMR (850 MHz, DMSO-d_6_): δ 0.93 (s, 3H, CH_3_), 1.01 (s, 3H, CH_3_), 2.11 (dd, 2H, *J* = 16.00 Hz, H-8a and H-8b), 2.31 (dd, 2H, *J* = 16.4 Hz, H-6a and H-6b), 2.39 (S, 3H, CH_3_), 4.45 (s, 1H, C4 –H), 6.41 (br’s, 2H, NH_2_, D_2_O exchangeable), 7.21–7.46 (m, 8H, ArH), 7.53–7.74 (m, 2H, Ar H), 7.85 (d, 2H, *J* = 7.8 Hz, ArH), 9.29 (br’s, 1H, NH, D_2_O exchangeable). ^13 ^C NMR (213 MHz, CDCl_3_): δ 19.2, 27.1, 29.6, 32.4, 36.2, 40.1, 50.7, 80.9, 115.1, 127.9, 128.3, 129.8, 129.9, 131.6, 133.4, 138.6, 140.0, 148.6, 151.1, 168.4, 190.3, 196.8; MS *m/z* (%): 605 (M^+^, 29.6), 607 (M^+^+2, 29.5). Anal. for C_3z 1_H_29_BrN_2_O_4_S: C, 61.49; H, 4.83; N, 4.63; S, 5.29. found: C, 61.68; H, 4.79; N, 4.55; S, 5.25%.

### 2.3. Biological evaluation

#### 2.3.1. *In vitro* cytotoxicity

MTT assay was used to evaluate the *in vitro* cytotoxicity of the new compounds against breast cancer cell lines MCF-7^38^. More details are present in the Supplementary file.

#### 2.3.2. Kinase assays

The activities of the examined compounds against EGFR, HER2, PDGFR-b, and VEGFR2 were *in vitro* tested using Abcam’s Human In cell ELISA Kit (ab 126419) for EGFR, ADP-Glo TM Kinase Assay for Her2, PDGFR-b, Active, Recombinant protein expressed in Sf9 cells for VEGFR2 (KDR) Kinase Assay Kit Catalog #40325, respectively. The procedure of the used kits was done according to the manufacturer, s instructions.

#### 2.3.3. Cell cycle analysis and apoptosis detection

Cell cycle analysis and apoptosis investigation were carried out by flow cytometry.^39^^,^^40^ MCF-7 cells were seeded at 8 × 104 and incubated at 37 °C, 5% CO_2_ overnight. After treatment with the tested compound, for 24 h, cell pellets were collected and centrifuged (300 g, 5 min).

### 2.4. Molecular docking method

The docking studies had been calculated and studies using Molecular Operating Environment (MOE, 2019.0102) software^41–43^. All minimisations were performed with MOE until an RMSD gradient of 0.1 kcal·mol^−1 ^Å^−1^ with MMFF94x force field and the partial charges were automatically calculated.

#### 2.4.1. Human epidermal growth factor receptor 2 (HER2)

The X-ray crystallographic structure of human HER2 (HER2) co-crystalised with 2-{2-[4-({5-chloro-6-[3-(trifluoromethyl)phenoxy]pyridin-3-yl}amino)-5H-pyrrolo[3,2-d]pyrimidin-5-yl]ethoxy}ethanol (03Q) (PDB ID: 3PP0) was downloaded from the protein data bank (https://www.rcsb.org/structure/3PP0). For each co-crystallised enzyme, water molecules and ligands which are not involved in the binding were removed. The protein was prepared for the docking study using *Protonate 3D* protocol in MOE with default options. The co-crystalised ligand (03Q) was used to define the binding site for docking. Triangle Matcher placement method and London dG scoring function were used for docking.

#### 2.4.2. Platelet-derived growth factor receptor α (PDGFRA)

The X-ray crystallographic structure of human platelet-derived growth factor receptor α (PDGFRA) co-crystalised with Imatinib (PDB ID: 6JOL) was downloaded from the protein data bank (https://www.rcsb.org/structure/6JOL). For each co-crystallised enzyme, water molecules and ligands which are not involved in the binding were removed. The protein was prepared for the docking study using *Protonate 3D* protocol in MOE with default options. The co-crystalised ligand (imatinib) was used to define the binding site for docking. Triangle Matcher placement method and London dG scoring function were used for docking.

## 3. Results and discussion

### 3.1. Chemistry

**Scheme 1.**
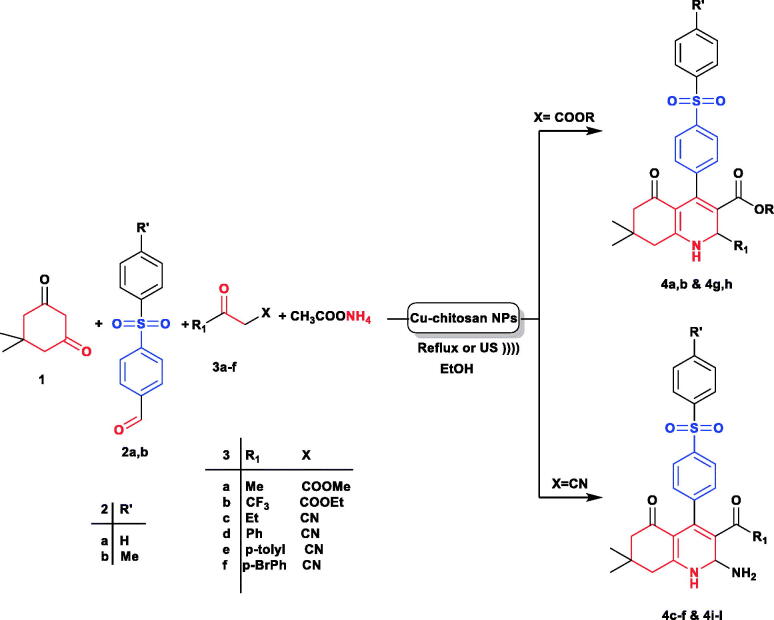
Synthesis of novel tetrahydroquinolin-5(1H)-ones **4a–l.**

We synthesised a series of 12 compounds bearing 4,6,7,8-tetrahydroquinolin-5(1H)-one scaffold according **to** Scheme 1. Compounds **4a–l** were synthesised following the previously reported optimised multicomponent green reaction procedures^35^ by treating dimedone (**1**) and aldehydes **2a, b** namely; 4-(phenylsulfonyl)benzaldehyde and 4-tosylbenzaldehyde respectively with various active methylene compounds **3a–f** in the presence of ammonium acetate in ethanol under ultrasonic irradiation conditions using chitosan decorated copper nanoparticles (CS/CuNPs) as a catalyst.

The progress of reactions was monitored by TLC till the disappearance of reactants. The beneficial effect of ultrasound is noticeable over conventional conditions on this reaction (Table 1), reducing the reaction time from 5 to 6 h into 20–30 min. and the yield of reaction increased to 90–95% under ultrasound irradiation compared to 78–84% under conventional conditions (Table 1).

The reaction products were identified as the polysubstituted 4,6,7,8-tetrahydroquinolin-5(1H)-ones **4a–l** in all cases based on its ^1^–H NMR spectrum. For example, the ^1^H NMR spectrum of the isolated reaction product **4a** revealed five singlet signals at δ 0.80, 0.98, 2.28, 3.49, and 4.91 ppm due to the four methyl groups and CH-4, respectively, two shielded doublets (dd) at δ 1.97, 2.15, ppm with coupling constants of approximately 16 Hz due to the H8a, H8b, another two shielded doublets (dd) at δ 2.33, and 2.41 ppm with coupling constant of 16.4 Hz due to H6a, H6b and two doublets and aromatic multiplet in the region 7.76–7.80 ppm due to 9 aromatic protons. In addition to a D_2_O exchangeable signal at 9.21 indicates the presence of NH group. Also, the ^13 ^C NMR of the isolated product **4a** adds strong evidence for the proposed structure, which agrees with the structure formed as shown in Scheme 1 (*cf.* experimental part).

The apparent effect of ultrasound on the reaction mentioned above may be attributed to the fact that ultrasonic irradiation gives the reactants sufficient energy to exceed the energy barrier of the reaction, thus, 4,6,7,8-tetrahydroquinolin-5(1H)-ones **4a–l** formed. This sufficient energy can be reasonably interpreted in terms of the physical phenomenon called acoustic cavitation (in our case, at solid-liquid interfaces). The most accepted proposed mechanism for the effect of cavitation near surfaces^44^^,^^45^ is microjet impact and shockwave damage. Along with the shock wave associated with the cavitation collapse, the jet causes localised deformation and surface erosion, which increases the possible reaction area. Therefore, the treated surfaces contain an increased number of dislocations that are widely considered to be the active sites in catalysis.

### 3.2. Biological evaluation

#### 3.2.1. *In vitro* anticancer activity

This work represents the construction of a new series of compounds bearing 7,7-dimethyl-4–(4-(phenylsulfonyl)phenyl)-4,6,7,8-tetrahydroquinolin-5(1*H*)-one scaffold **4a–l** of potential anti-breast cancer activity. To evaluate the cytotoxic activity of the new derivatives, they were subjected to MTT cell viability assay against human breast cancer MCF-7 cell line utilising staurosporine as a reference drug.^46–48^ The obtained data were represented as IC_50_ (µM) values which are the average of at least three independent experiments and tabulated in Table 2.

**Table 2.** *In vitro* cell cytotoxic activity of the new compounds against MCF-7 cancer cells.

Compound No		Anticancer activity against MCF-7 cells IC_50_ (µM) mean ± SD
**4a**	Ar = phenyl; R = OCH_3_; R^’^ = CH_3_	0.103 ± 0.013
**4b**	Ar = phenyl; R = OC_2_H_5_; R^’^ = CF_3_	0.002 ± 0.001
**4c**	Ar = phenyl; R = OC_2_H_5_, R^’^ = NH_2_	0.015 ± 0.002
**4d**	Ar, R = phenyl; R^’^ = NH_2_	0.007 ± 0.001
**4e**	Ar, R = *p*-tolyl; R^’^ = NH_2_	0.004 ± 0.002
**4f**	Ar = phenyl; R = *p*-Br-phenyl; R^’^ = NH_2_	0.027 ± 0.014
**4g**	Ar = *p*-tolyl; R = OCH_3_; R^’^ = CH_3_	0.027 ± 0.003
**4h**	Ar = *p*-tolyl; R = OC_2_H_5_; R^’^ = CF_3_	0.041 ± 0.006
**4i**	Ar = *p*-tolyl; R = OC_2_H_5_; R^’^ = NH_2_	0.023 ± 0.003
**4j**	Ar = *p*-tolyl; R = phenyl; R^’^ = NH_2_	0.003 ± 0.005
**4k**	Ar, R = *p*-tolyl; R^’^ = NH_2_	0.004 ± 0.001
**4l**	Ar = *p*-tolyl; R = *p*-Br-phenyl; R^’^ = NH_2_	0.007 ± 0.001
**Staurosporine**		0.005 ± 0.002

The new derivatives showed variable degrees of cytotoxic activities. Interestingly, the 2-trifluoromethyl derivative **4b** exhibited about 2.5-fold more potent activity than the standard drug staurosporine (IC_50_; 0.002 µM, IC_50staurosporine_; 0.005 µM). The potent cytotoxic effect of **4b** could be explained due to the electron-withdrawing power of fluorine atom, alongside the increased carbon–fluorine bond energy, that significantly potentiates the metabolic stability of the host molecule and enhances its lipophilicity facilitating the cell membrane permeation and its pronounced cytotoxic effect^38^. A slight decrease in the potency was detected upon replacement of –CF_3_ group by NH_2_ as compounds **4j, 4e, 4k** exhibiting about 1–1.6-fold more potent activity than that resulted by staurosporine of IC_50_ values of 0.003, 0.004, and 0.004 µM, respectively. Further decrease in the inhibitory activity to be 1.4-fold less potent than the standard drug was obtained upon replacement of the 3-methoxy/ethoxy groups by phenyl or *p*-bromophenyl groups as compounds **4d**, **4 l**, of IC_50_; 0.007 µM. The rest of the compounds produced a detectable decrease in the cytotoxic potency of IC_50,_ ranging from 0.023 to 0.103 µM. It could be noted that the significant increase in the hydrophobicity of the compounds negatively affects the cytotoxic activity of the new tetrahydroquinolinone derivatives.

#### 3.2.2. The effect of compounds 4b, 4j, 4k, and 4e against the normal WI38 cells

One of the characteristics differentiating different anticancer agents from each other is the recurrence and severity of their side effects to the normal cells at their therapeutic doses. Accordingly, the safety profile of the most promising candidates (**4b, 4j, 4k,** and **4e)** was evaluated against the normal WI38 cells derived from lung tissues in comparison with staurosporine as a reference drug-using MTT assay^46–48^ (Table 3). It is worthy of mentioning that the IC_50_ values of the target compounds **4j**, **4b** against the normal WI38 cells were 20 and 24-fold higher than their IC_50_ doses against the cancer cells and about 3-fold higher than the IC_50_ value of reference drug staurosporine confirming the promising safety profile of both compounds. Whereas, less safety profile was investigated by compounds **4e** and **4k** producing IC_50_ values against WI38 cells that were only 4-fold higher than their IC_50_ doses against MCF-7 cells and approximately equal to that obtained by staurosporine (Table 2).

**Table 3.** The effect of some compounds as representative examples against the normal WI38 cells.

Compound No.	IC_50_ (µM) mean ± SD
**4e**	0.0149 ± 0.003
**4j**	0.048 ± 0.008
**4b**	0.045 ± 0.013
**4k**	0.0176 ± 0.009
**Staurosporine**	0.013 ± 0.002

#### 3.2.3. *In vitro* kinase inhibition assay

To explore the mechanistic insight into the cytotoxic potentials of the new quinolone compounds, *in vitro* kinase assay was performed to evaluate the kinase suppression activity of the most promising cytotoxic candidates **4b**, **4j** against four different RTK. Which are: EGFR, human epidermal growth factor receptor (HER2), PDGFR-α, and VEGFR-2. It has been detected that compound **4j** was more potent than **4b** as EGFR inhibitor with IC_50_ values of 0.07 × 10^−3^ and 0.11 × 10^−3 ^µM, respectively, but both were less potent than the Sorafenib of IC_50_ 0.04 × 10^−3 ^µM (Table 4). On the other hand, compound **4j** exhibited a significant inhibitory effect against HER-2, which is about 1.6 folds more potent than the Sorafenib of IC_50_; 0.17 × 10^−3 ^µM, IC_50sorafenib_; 0.28 × 10^−3 ^µM. Also, compound **4j** notably inhibited PDGFR-α by 1.9-fold more than the reference sorafenib of IC_50_; 0.07 × 10^−3^, 0.13 × 10^−3 ^µM, respectively. Furthermore, both compounds **4b** and **4j** revealed VEGFR-2 inhibitory effect of about 6.2 and 1.8-fold less than that of Sorafenib of IC_50_ values 1.05, 0.30 × 10^−3 ^µM, IC_50sorafenib_; 0.17 × 10^−3 ^µM, respectively. Furthermore, the obtained data revealed the distinct inhibitory profile of the amino tetrahydroquinoline derivative **4j** in comparison with the reference drug sorafenib as illustrated in Table 3.

**Table 4.** Protein kinase inhibition of compounds **4b** and **4j** in comparison with Sorafenib.

Compound No	EGFR IC_50_ µM × 10^−3^	HER-2 IC_50_ µM × 10^−3^	PDGFR-α IC_50_ µM × 10^−3^	VEGFR-2 IC_50_ µM × 10^−3^
**4b**	0.11 ± 0.03	0.30 ± 0.05	0.21 ± 0.04	1.05 ± 0.02
**4j**	0.07 ± 0.02	0.17 ± 0.02	0.07 ± 0.01	0.30 ± 0.06
**Sorafenib**	0.04 ± 0.02	0.28 ± 0.04	0.13 ± 0.02	0.17 ± 0.02

#### 3.2.4. Apoptosis assay

**Figure 4.**
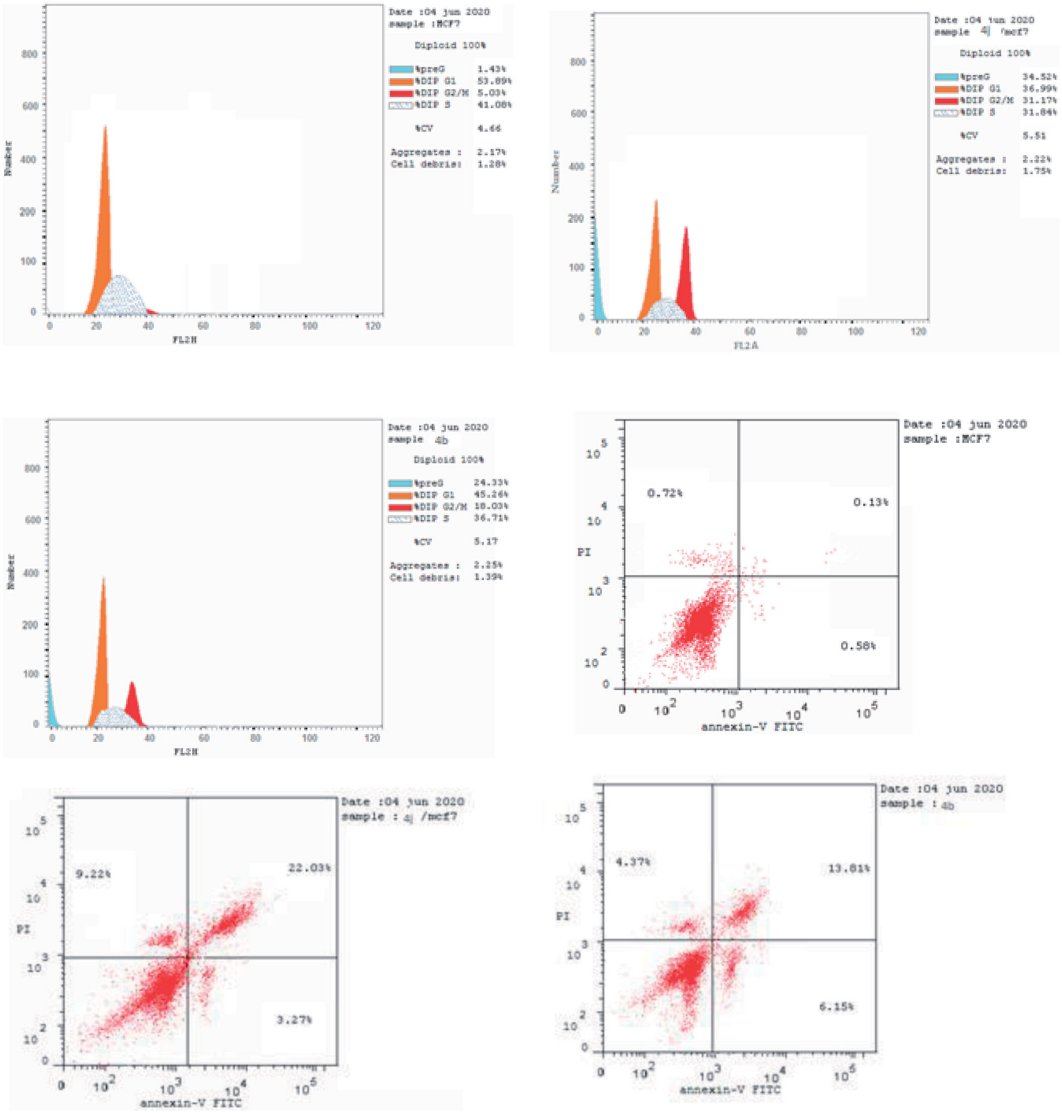
Apoptotic effects of compounds **4b** and **4j** against MCF-7 cells.

**Figure 5.**
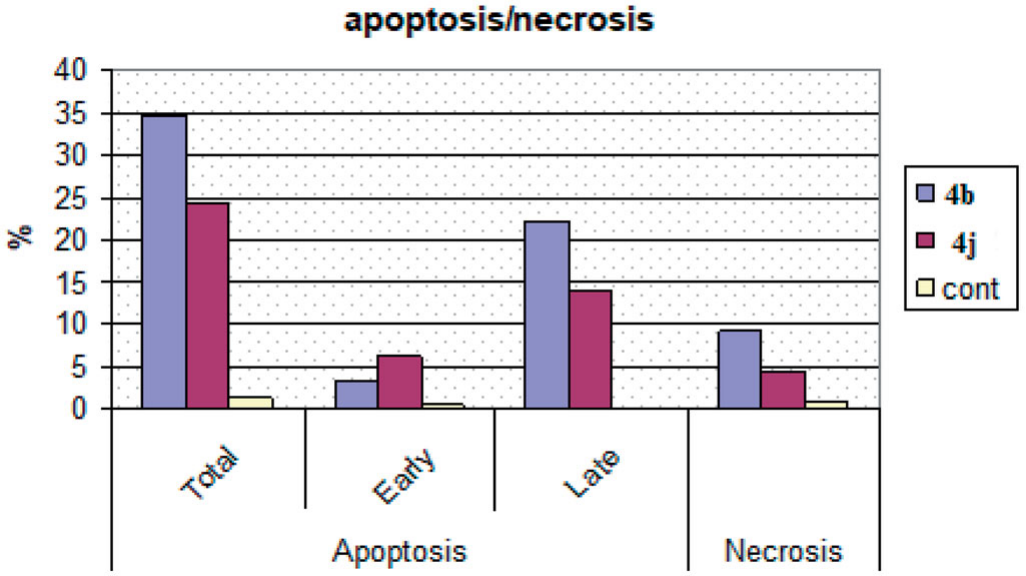
Apoptosis induction against MCF-7 cells caused by the derivatives **4b, 4j**.

The derivatives **4b** and **4j** were selected to study their apoptotic effects on MCF-7 cancer cells using Annexin-V/PI binding assay based on their promising cytotoxic potency and various kinase inhibitory effects. Staining MCF-7 cells was carried out with the two dyes; Annexin V/propidium iodide (PI) after treating them with compounds **4b** and **4j** at their IC_50_ concentrations of 0.002 and 0.003 µM for 24 h. Flow cytometry method^39^^,^^49^ has been used to detect the corresponding red (PI) and green (FITC) fluorescence. It has been noted that there was an increment in the percentages of the late apoptosis produced by the evaluated compounds **4b** and **4j** from 0.13% (control DMSO/MCF-7 cells) to 13.81% and 22.03%, respectively. Also, the tested compounds produced early apoptotic effects of 6.15% and 3.27% compared to 0.58% of the untreated MCF-7 cell with necrosis percent of 4.37% and 9.22%, respectively, *vs.* 0.66% produced by the DMSO control (Figures 4 and 5).

The proportion of the late apoptosis produced by both **4b** and **4j** was higher than the proportion of the early phase, which makes recovering the dead cells to safe ones is more challenging.

#### 3.2.5. Cell cycle analysis

**Figure 6.**
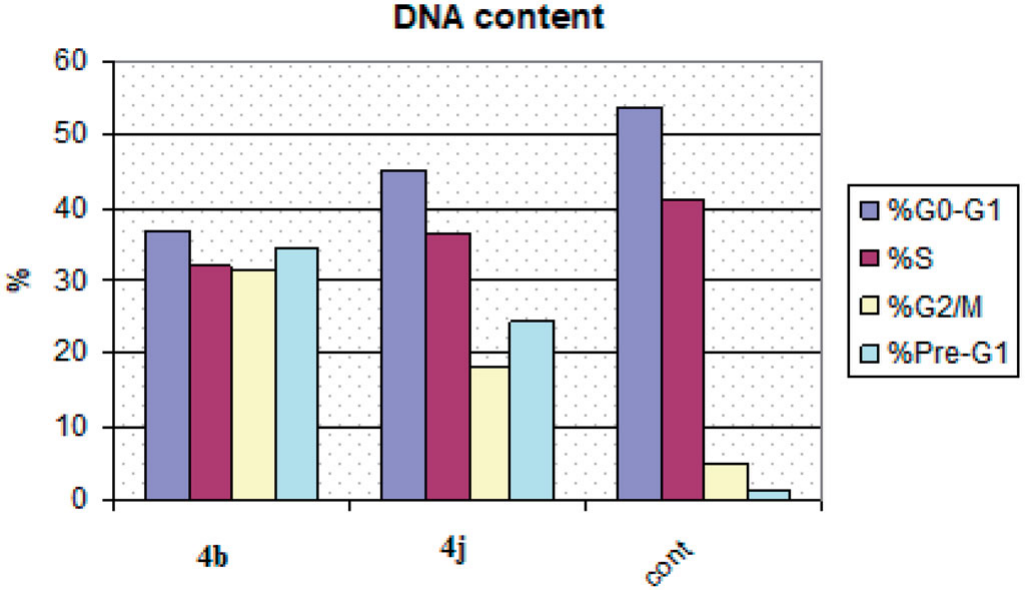
Cell Cycle analysis results of compounds **4b** and **4j**.

The induction of apoptosis is one of the most crucial tools that confirm the effectiveness of cancer therapy^50^. Cell cycle checkpoints are G1 (restriction or start), S (metaphase), and G2/M. One of the main functions of the anticancer therapeutics is stoppage of the cell division at these checkpoints.^51^ Thus, MCF-7 cells were incubated with compounds **4b** and **4j** at their IC_50_ concentrations (0.002, 0.003 µM) for 24 h. The cells were stained with Annexin V/PI and examined using flow cytometry procedure. The resultant data revealed that there was cell accumulation of percentages 24.33% and 18.03% at pre G1 and G2/M phases in MCF-7 cells treated with compound **4b** and cell accumulation percentages of 34.52% and 31.17% at pre G1 and G2/M phases in MCF-7 cells treated with compound **4j** comparing to 1.43% and 5.03% of the untreated MCF-7 cells. This result represents that there was cell cycle arrest at G2/M phase with mitotic cycle cessation (Table 5, Figure 6).

**Table 5.** Cell cycle analysis of compounds **4b** and **4j.**

Compound No	%G0–G1	%S	%G2/M	%Pre-G1
**4b**	45.26	36.71	18.03	24.33
**4j**	36.99	31.84	31.17	34.52
**Cont. MCF-7**	53.89	41.08	5.03	1.43

## 4. Molecular docking study

The molecular modelling studies were carried out using MOE (2019.0102) software. All minimisations were performed with MOE until an RMSD gradient of 0.1 kcal·mol^−1 ^Å^−1^ with MMFF94x force field, and the partial charges were automatically calculated.

### 4.1. Human epidermal growth factor receptor 2 (HER2)

The X-ray crystallographic structure of human HER2 (HER2) co-crystalised with 2-{2-[4-({5-chloro-6-[3-(trifluoromethyl)phenoxy]pyridin-3-yl}amino)-5H-pyrrolo[3,2-d]pyrimidin-5-yl]ethoxy}ethanol (03Q) (PDB ID: 3PP0) was downloaded from the protein data bank (https://www.rcsb.org/structure/3PP0). For each co-crystallised enzyme, water molecules and ligands that are not involved in the binding were removed. The protein was prepared for the docking study using *Protonate 3D* protocol in MOE with default options. The co-crystalised ligand (03Q) was used to define the binding site for docking. Triangle Matcher placement method and London dG scoring function were used for docking.

**Figure 7.**
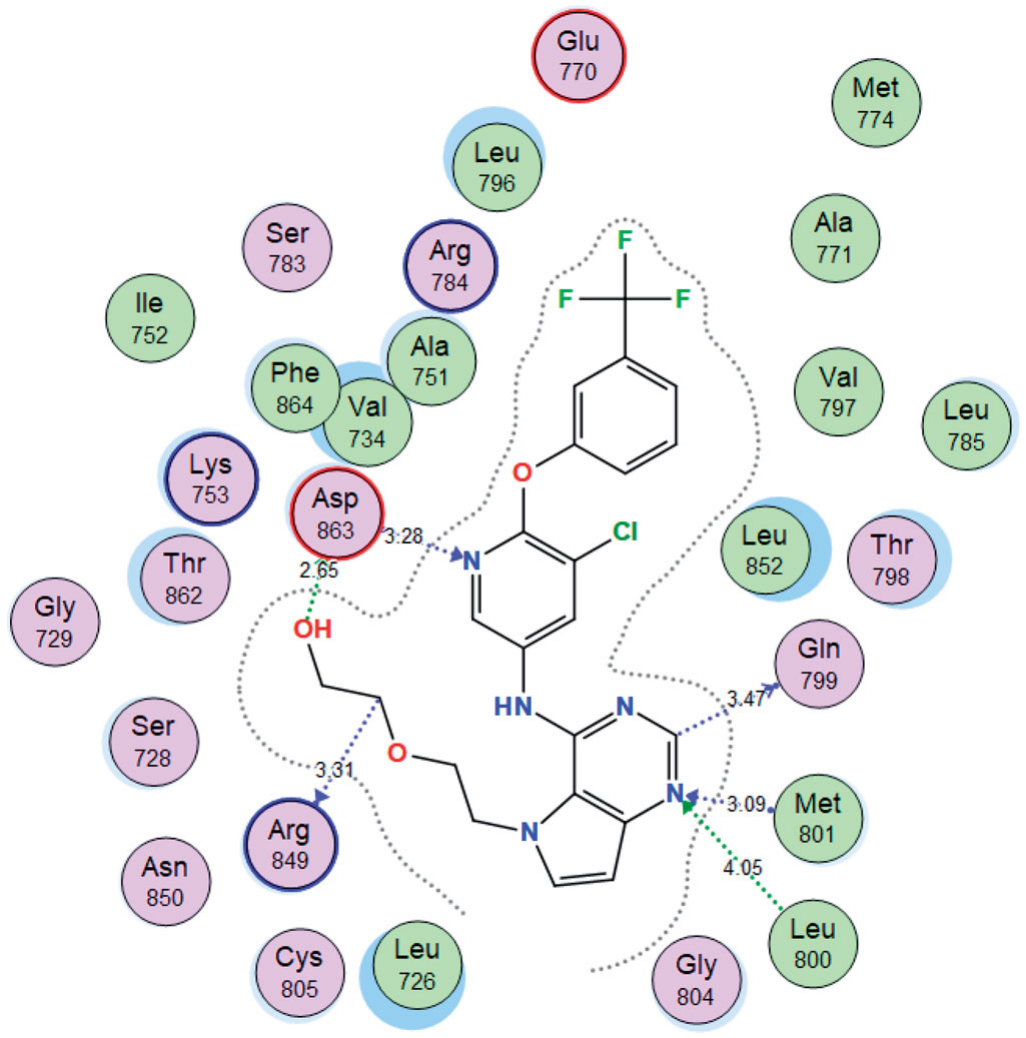
2D interactions of 03Q within HER2 active site.

Through examination of the binding interactions of 03Q to the active site of the enzyme, it shows strong bond interactions with Gln799, Leu800, Met801, Arg849, and Asp863 (Figure 7).

**Figure 8.**
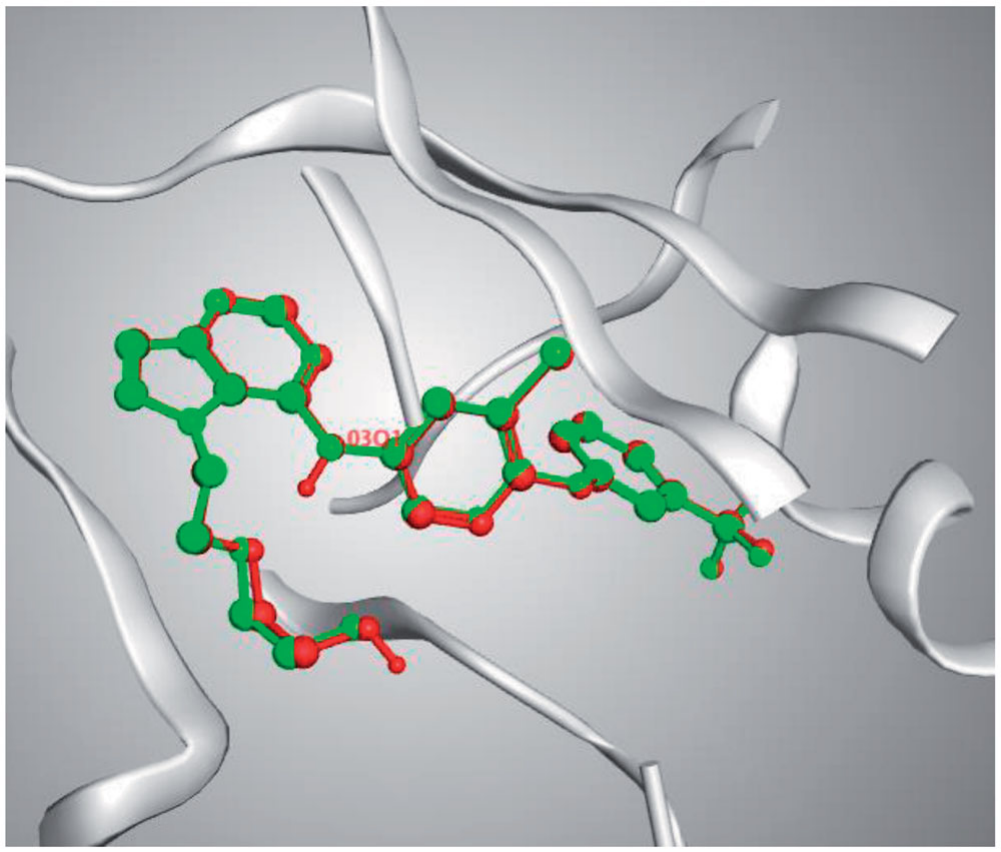
3D representation of the superimposition of the co-crystallised (red) and the docking pose (green) of 30Q in the active site of HER2.

The docking setup was first validated by self-docking the co-crystallised ligand (30Q) in the vicinity of the enzyme’s binding site. The docking score (S) was −17.1413 kcal/mol, and the root means square deviation (RMSD) was 0.14339 Å (Figure 8).

**Figure 9.**
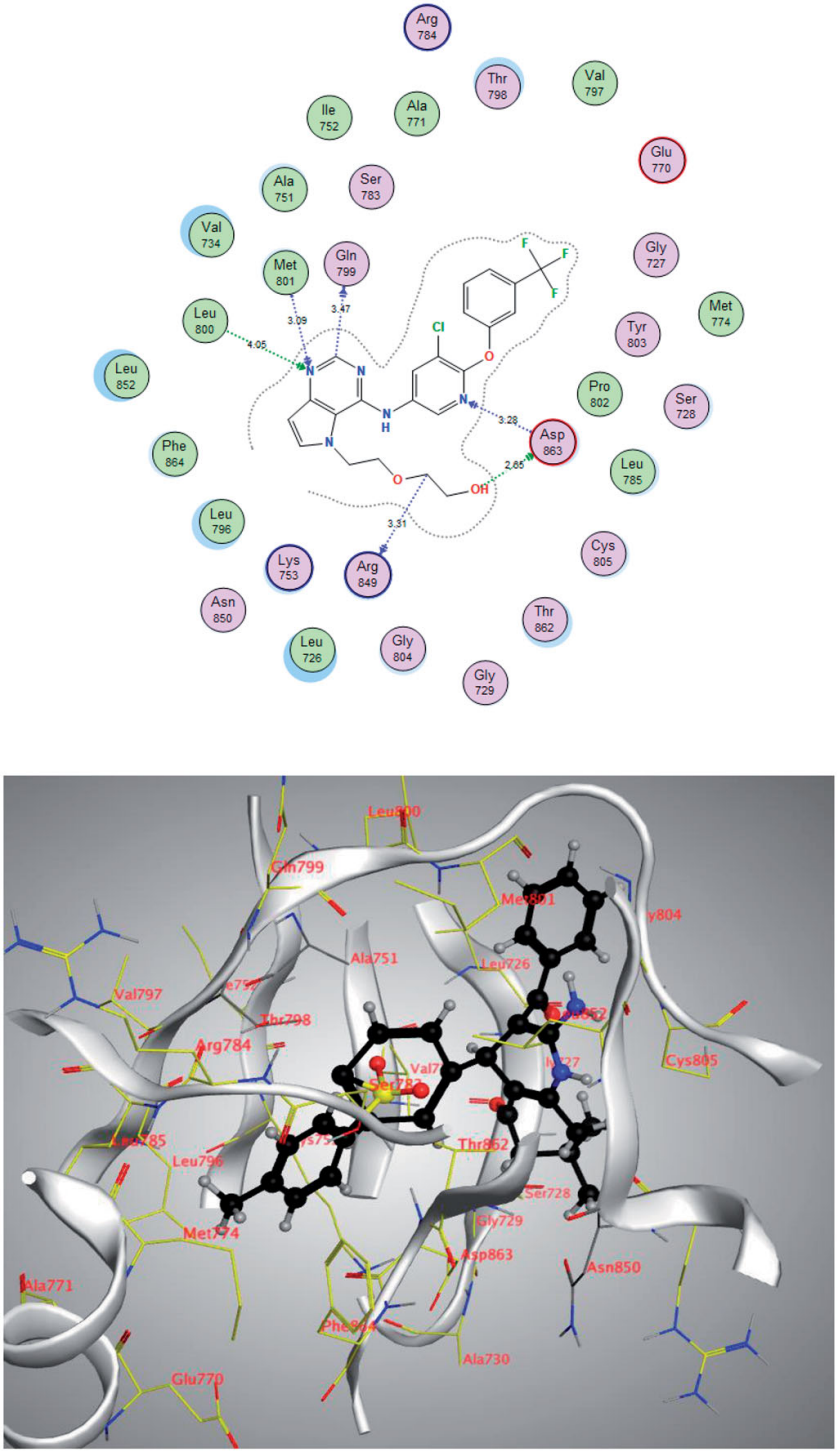
2D and 3D diagram of compound **4j** interactions within HER2 binding site.

**Figure 10.**
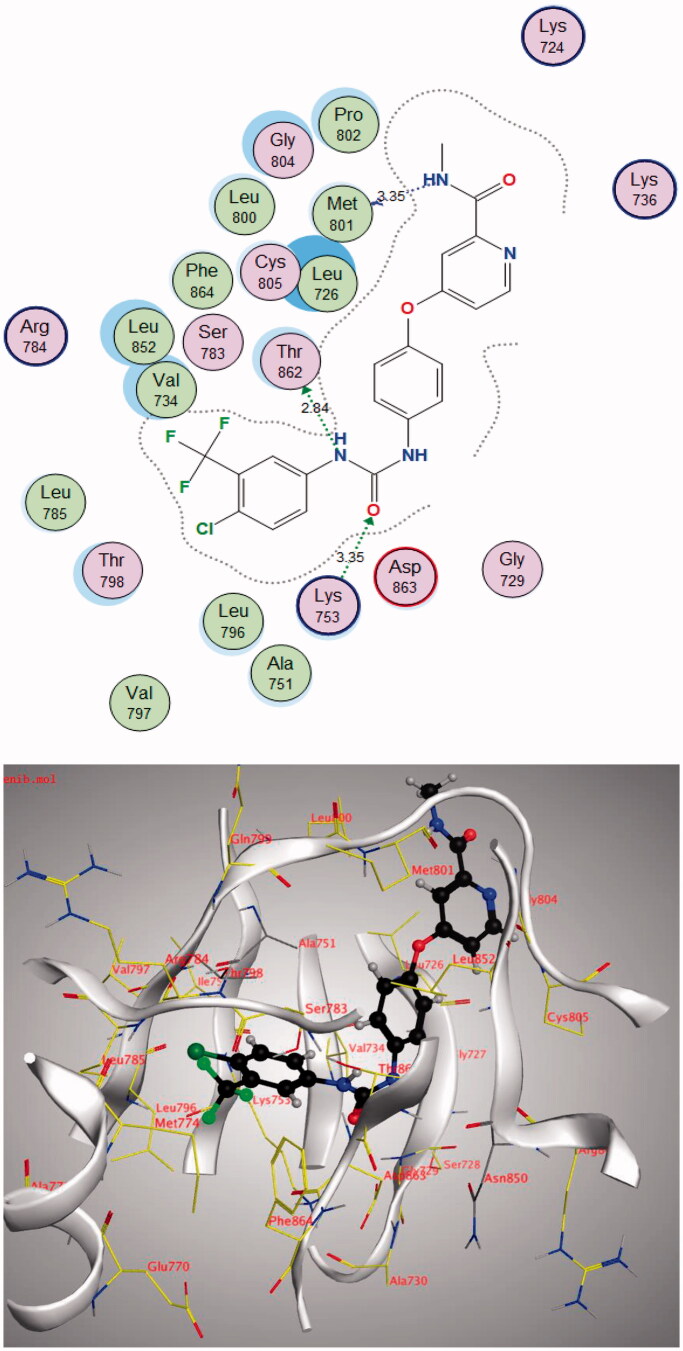
2D and 3D diagram of compound Sorafenib interactions withing HER2 binding site.

The **4j** compound showed high energy binding score (−17.3597 kcal/mol.) similar to that of the co-crystallised ligand and higher than Sorafenib. Moreover, it showed good binding interactions with the amino acids in the active site of the receptor. The results are summarised in Table 6 and Figures 9 and 10.

**Table 6.** Docking data of compound **4j** in the active site of HER2.

Compound	S (kcal/mol)	Amino acids	Interacting groups	Type of interaction	Length
**4j**	−17.3597	Gln799	CH (Pyrimidine)	Electrostatic	3.47
		Leu800	N (Pyrimidine)	H-bond acceptor	4.05
		Met801	N (Pyrimidine)	H-bond acceptor	3.09
		Arg849	CH_2_	Electrostatic	3.31.2021
		Asp863	OH	H-bond donor	2.65
		Asp863	N (Pyridine)	H-bond acceptor	3.28
**Sorafenib**	−15.4085	Lys753	O (C = O)	H-bond acceptor	3.35
		Met801	NH (Amide)	H-bond donor	3.25
		Thr862	NH (Urea)	H-bond donor	2.84

### 4.2. Platelet-derived growth factor receptor α (PDGFr-α)

The X-ray crystallographic structure of human PDGFRA co-crystalised with Imatinib (PDB ID: 6JOL) was downloaded from the protein data bank (https://www.rcsb.org/structure/6JOL). For each co-crystallised enzyme, water molecules and ligands that are not involved in the binding were removed. The protein was prepared for the docking study using *Protonate 3D* protocol in MOE with default options. The co-crystalised ligand (imatinib) was used to define the binding site for docking. Triangle Matcher placement method and London dG scoring function were used for docking.

**Figure 11.**
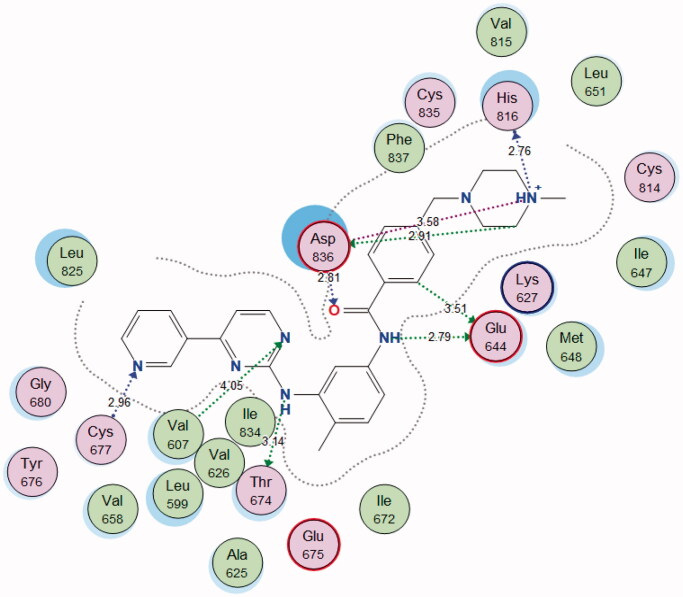
2D interactions of imatinib within PDGFR-α active site.

By examining the binding interactions of imatinib to the enzyme’s active site, it shows strong bond interactions with Val607, Glu644, Thr674, Cys677, His816, and Asp836 (Figure 11).

**Figure 12.**
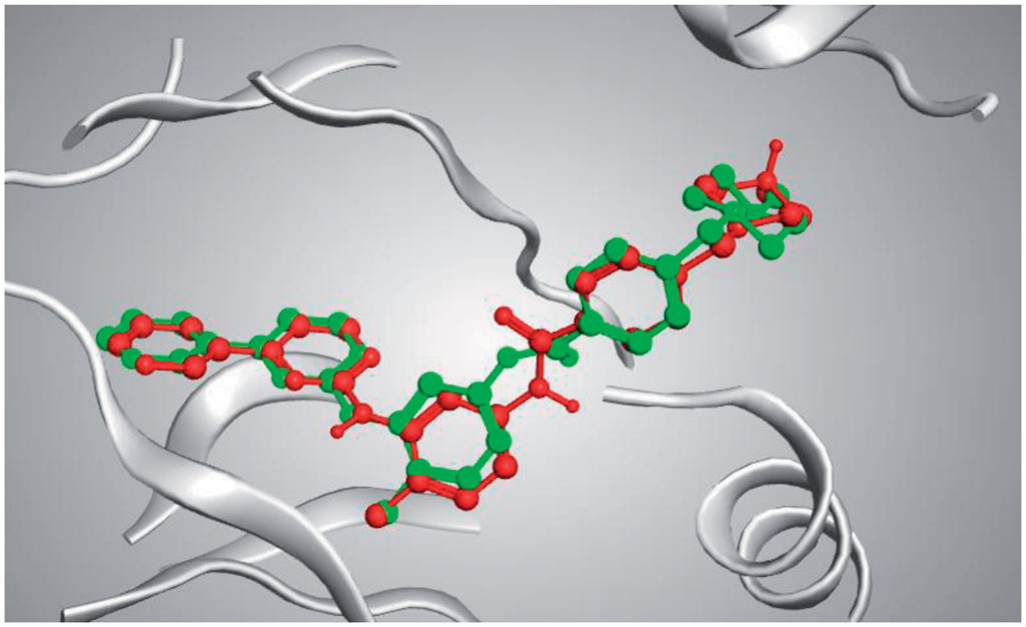
3D representation of the superimposition of the co-crystallised (red) and the docking pose (green) of imatinib in the active site of PDGFR-α.

The docking setup was first validated by self-docking the co-crystallised ligand (imatinib) in the vicinity of the enzyme’s binding site. The docking score (S) was −18.0520 kcal/mol, and RMSD was 0.6983 Å (Figure 12).

**Figure 13.**
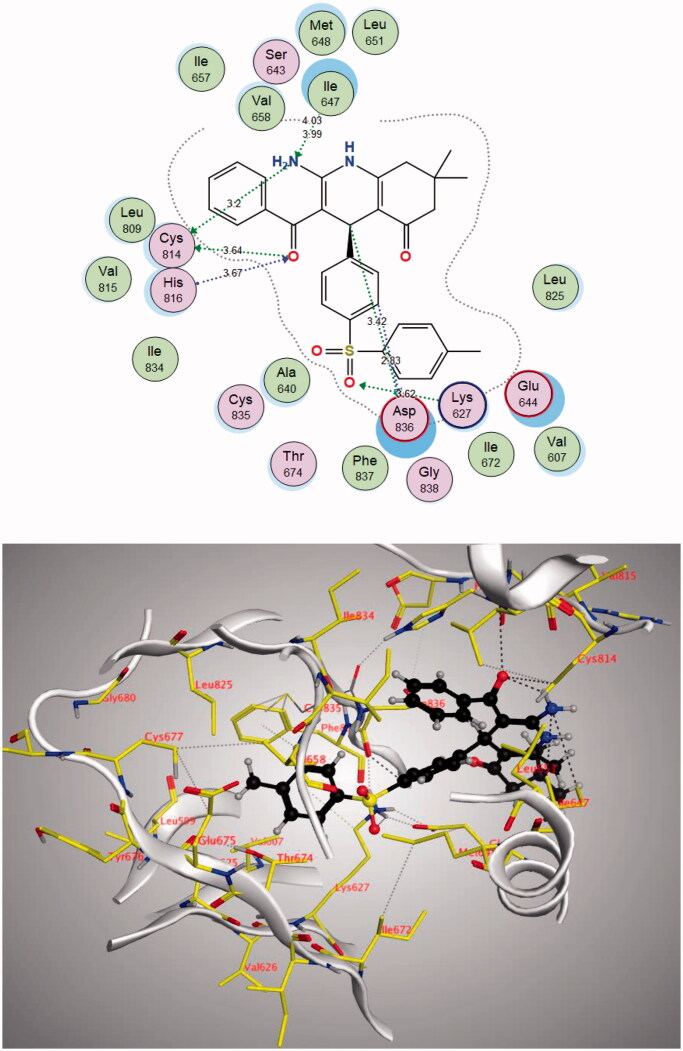
2D and 3D diagram of compound **4j** interactions within PDGFR-α binding site.

**Figure 14.**
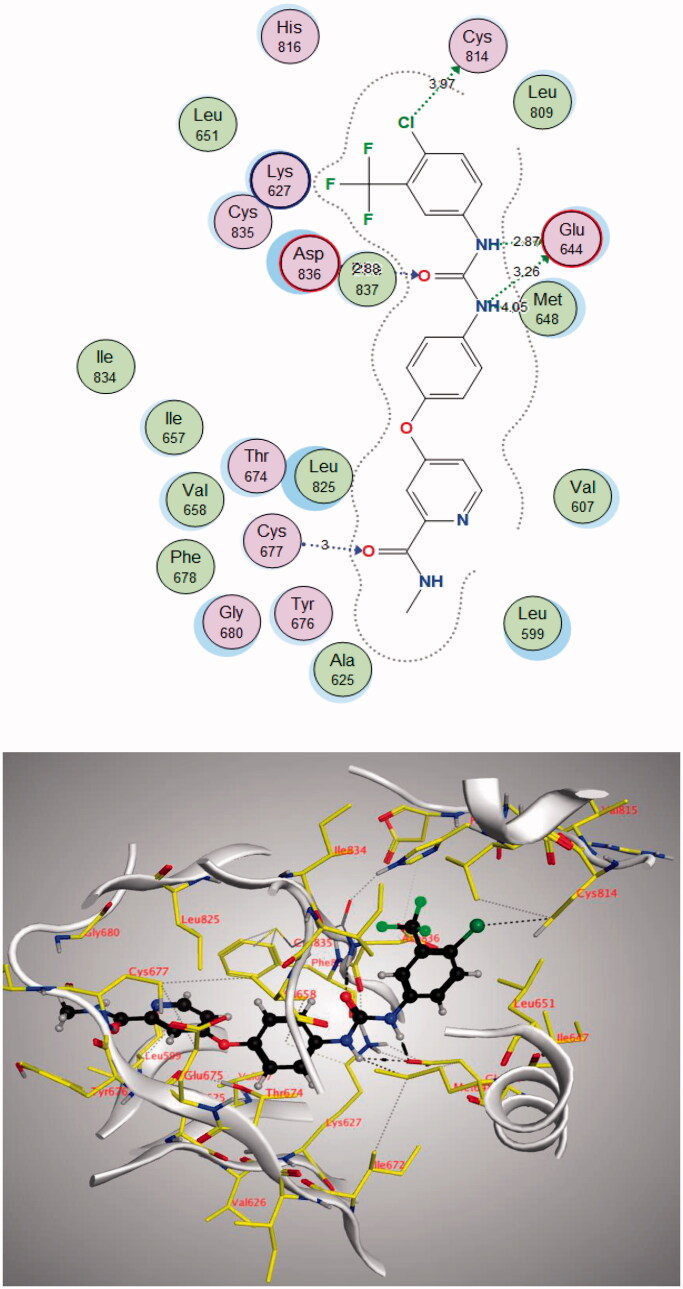
2D and 3D diagram of Sorafenib interactions within PDGFR-α binding site.

The **4j** compound showed a similar energy binding score to that of Sorafenib. It exhibited good binding interactions with the amino acid in PDGFR-α active site. The results are summarised in Table 7 and Figures 13 and 14.

**Table 7.** Docking results of compound **4j** in the active site of PDGFR-α.

Compound	S (kcal/mol)	Amino acids	Interacting groups	Type of interaction	Length
**4j**	−13.2171	Lys627	O (S = O)	H-bond acceptor	3.62
		Ile647	NH_2_	H-bond acceptor	3.99
		Cys814	NH_2_	H-bond donor	3.20
		Cys814	O (C = O)	Electrostatic	3.64
		His816	O (C = O)	H-bond acceptor	3.67
		Asp836	CH (Phenyl)	Electrostatic	2.83
		Asp836	CH	Electrostatic	3.42
**Sorafenib**	−13.0476	Glu644	NH	H-bond donor	2.87
		Glu644	NH	H-bond donor	3.26
		Met648	NH	H-bond acceptor	4.05
		Cys677	O (C = O)	H-bond acceptor	3.00
		Cys814	Cl	Halogen bond	3.97
		Asp836	O (C = O)	H-bond acceptor	2.88

## 5. Conclusion

A new class of 4,6,7,8-tetrahydroquinolin-5(1*H*)-one-based derivatives has been green synthesised as anti-breast cancer (MCF-7) agents of potential multi-targeting RTKs. The compounds **4a–l** were examined as cytotoxic agents against MCF-7 cancer cells using MTT assay utilising staurosporine as a standard drug. The compounds **4b**, **4e**, **4j**, and **4k** appeared as the most promising cytotoxic candidates. That revealing a more potent inhibiting effect than staurosporine, displaying IC_50_ values ranging from 0.002 to 0.004 µM *vs*. IC_50_ value of staurosporine, 0.007 µM. The safety profile of the latter derivatives was evaluated against the normal WI38 cells. The compounds **4b** and **4j** appeared as the safest agents on the normal cells. Furthermore, the compound **4b**, **4j** were selected as representative examples to evaluate their suppression activity against EGFR, HER-2, PDGFR-α, and VEGFR-2 protein kinases. The compound **4j** investigated potent multi-targeting inhibitory activity in comparison with Sorafenib. In addition, the biological evidence revealed that the compound **4j** caused a marked apoptotic degree with a necrosis percentage 4.2%, leading to cell cycle disruption at G2/M phase in MCF-7 cancer cells. Accordingly, the new derivatives bearing 4,6,7,8-tetrahydroquinolin-5(1*H*)-one scaffold could be considered primary nuclei for further structural optimisation to get more potent, selective, and safer anticancer candidates. A molecular docking study was found in complete agreement with the obtained experimental results.

## Supplementary Material

Supplemental Material
