# Supplementary material for: Design and green synthesis of novel quinolinone derivatives of potential anti-breast cancer activity against MCF-7 cell line targeting multi-receptor tyrosine kinases
Source: J Enzyme Inhib Med Chem. 2021 Jul 1;36(1):1454–71. doi: 10.1080/14756366.2021.1944126 (PMC8259865; doi:10.1080/14756366.2021.1944126)

Design and green synthesis of novel quinolinone derivatives of potential anti-breast cancer activity against MCF-7 cell line targeting multi-receptor tyrosine kinases

Mohamed Mokhtar[1,*], Khadijah S. Alghamdi[2], Nesreen. S. Ahmed[3], Dina Bakhotmah[1], Tamer S. Saleh[4,5]

[1]Chemistry Department, Faculty of Science, King Abdulaziz University, Jeddah 21589, Saudi Arabia, [2]Chemistry Department, Faculty of Science, Albaha University, Saudi Arabia, [3]Department of Therapeutic Chemistry, National Research Centre, Dokki, Cairo 12622, Egypt, [4]University of Jeddah, College of Science, Department of Chemistry, Jeddah, Saudi Arabia, [5]Green Chemistry Department, National Research Centre, Dokki, 12622, Giza, Egypt

Supporting Information

S1: In vitro cytotoxicity experiment

S2: Molecular Docking Study results

S3: ^{1}H and ^{13}C NMR Charts of the synthesized compounds

* Contact: Mohamed Mokhtar, mmoustafa@kau.edu.sa, Chemistry Department, Faculty of Science, King Abdulaziz University, Jeddah 21589, Saudi Arabia

S1: In vitro cytotoxicity experiment

MTT assay was used to evaluate the in vitro cytotoxicity of the new compounds against breast cancer cell lines MCF-7 [39]. MTT assay depends on the reduction of the soluble 3-(4,5-methyl-2-thiazolyl)-2,5-diphenyl-2H-tetrazolium bromide (MTT) into a blue purple formazan product, mainly by mitochondrial reductase activity inside the living cells. The cells used in cytotoxicity assay were cultured in RPMI 1640 medium supplemented with 10% fetal calf serum. Cells suspended in the medium (2×10^4 cells/mL) were plated in 96-well culture plates and incubated at 37 °C in a 5% CO_2 incubator. After 12 h, the test sample (2 µL) was added to the cells (2×10^4) in 96-well plates and cultured at 37◦C for 3 days. The cultured cells were mixed with 20 µL of MTT solution and incubated for 4 h at 37 °C. The supernatant was carefully removed from each well and 100 µL of DMSO were added to each well to dissolve the formazan crystals which were formed by the cellular reduction of MTT. After mixing with a mechanical plate mixer, the absorbance of each well was measured by a microplate reader using a test wavelength of 570 nm.

S2: Molecular docking study results:

Molecular docking study

All the molecular modeling studies were carried out using Molecular Operating Environment (MOE, 2019.0102) software. All minimizations were performed with MOE until an RMSD gradient of 0.1 $kcal \cdot mol^{-1} Å^{-1}$ with MMFF94x force field and the partial charges were automatically calculated.

HER2 (human epidermal growth factor receptor 2):

The X-ray crystallographic structure of human HER2 (**HER2**) co-crystalized with 2-{2-[4-({5-chloro-6-[3-(trifluoromethyl)phenoxy]pyridin-3-yl}amino)-5H-pyrrolo[3,2-d]pyrimidin-5-yl]ethoxy}ethanol **(03Q)** (**PDB ID: 3PP0**) was downloaded from the protein data bank (**https://www.rcsb.org/structure/3PP0**). For each co-crystallized enzyme, water molecules and ligands which are not involved in the binding were removed, the protein was prepared for the docking study using *Protonate 3D* protocol in MOE with default options. The co-crystalized ligand (**03Q**) was used to define the binding site for docking. Triangle Matcher placement method and London dG scoring function were used for docking.

Through examination of the binding interactions of **03Q** to the active site of the enzyme, it shows strong bond interactions with Gln799, Leu800, Met801, Arg849 and Asp863 (**Figure 1**).

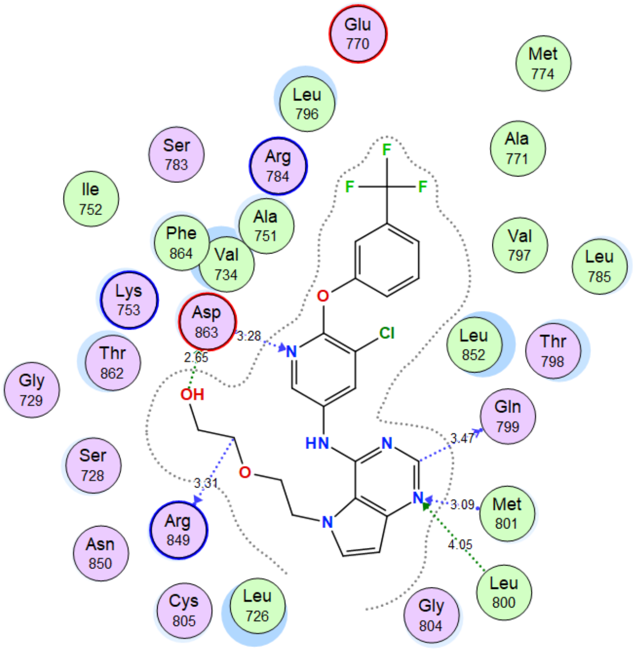

Figure 1: 2D interactions of **03Q** within **HER2** active site

Docking setup was first validated by self-docking of the co-crystallized ligand (**30Q**) in the vicinity of the binding site of the enzyme, the docking score (S) was -17.1413 kcal/mol. and root mean square deviation (RMSD) was 0.14339 Å (**Figure 2**).

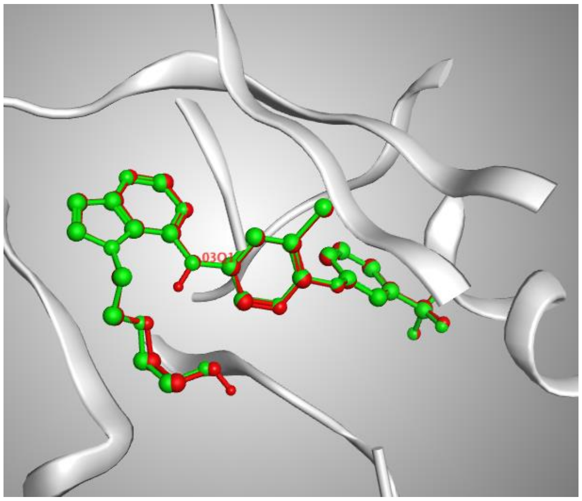

Figure 2: 3D representation of the superimposition of the co-crystallized (red) and the docking pose (green) of **30Q** in the active site of **HER2**

The 4j compound showed high energy binding score (-17.3597 kcal/mol.) similar to that of the co-crystallized ligand and higher than Sorafenib. Moreover, it showed good binding interactions with the amino acids in the active site of the receptor. The results are summarized in **Table (1)** & **Figures (3 & 4).**

Table (1): Docking results

Compound	S (kcal/mol)	Amino acids	Interacting groups	Type of interaction	Length

**4j**	-17.3597	Gln799	CH (Pyrimidine)	Electrostatic	3.47
		Leu800	N (Pyrimidine)	H-bond acceptor	4.05
		Met801	N (Pyrimidine)	H-bond acceptor	3.09
		Arg849	CH_2	Electrostatic	3.31.2021
		Asp863	OH	H-bond donor	2.65
		Asp863	N (Pyridine)	H-bond acceptor	3.28
**Sorafenib**	-15.4085	Lys753	O (C=O)	H-bond acceptor	3.35
		Met801	NH (Amide)	H-bond donor	3.25
		Thr862	NH (Urea)	H-bond donor	2.84

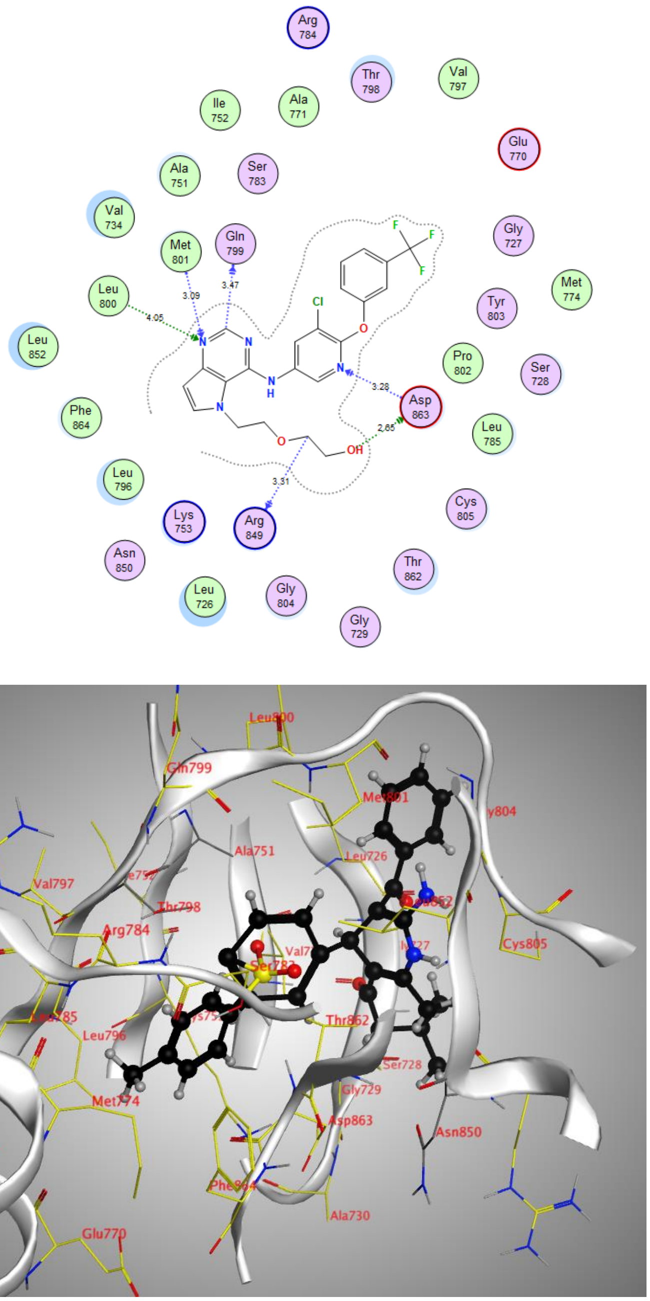

Figure 3: 2D & 3D diagram of compound **4j** interactions withing **HER2** binding site

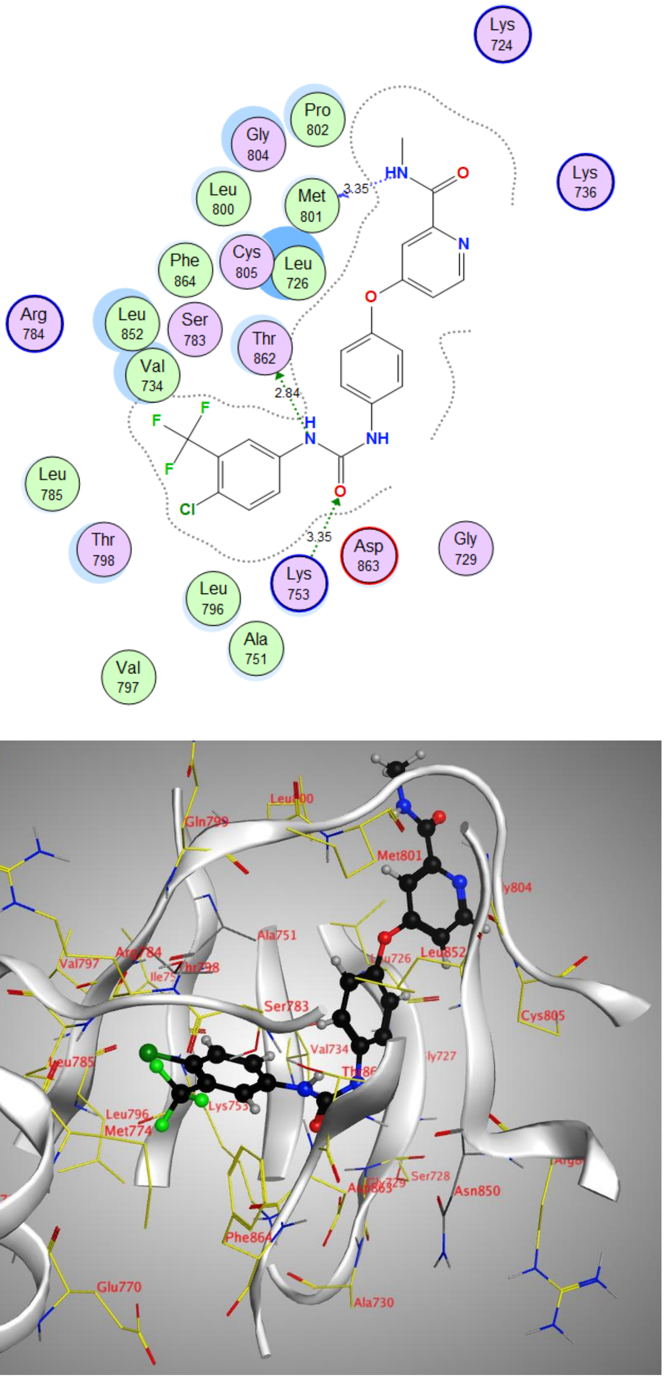

Figure 4: 2D & 3D diagram of compound **Sorafenib** interactions withing **HER2** binding site

PDGFRA (Platelet-Derived Growth Factor Receptor α)

The X-ray crystallographic structure of human Platelet-Derived Growth Factor Receptor α (**PDGFRA**) co-crystalized with **Imatinib** (**PDB ID: 6JOL**) was downloaded from the protein data bank (**https://www.rcsb.org/structure/6JOL**). For each co-crystallized enzyme, water molecules and ligands which are not involved in the binding were removed, the protein was prepared for the docking study using *Protonate 3D* protocol in MOE with default options. The co-crystalized ligand (**imatinib**) was used to define the binding site for docking. Triangle Matcher placement method and London dG scoring function were used for docking.

Through examination of the binding interactions of **imatinib** to the active site of the enzyme, it shows strong bond interactions with Val607, Glu644, Thr674, Cys677, His816 and Asp836 (**Figure 5**).

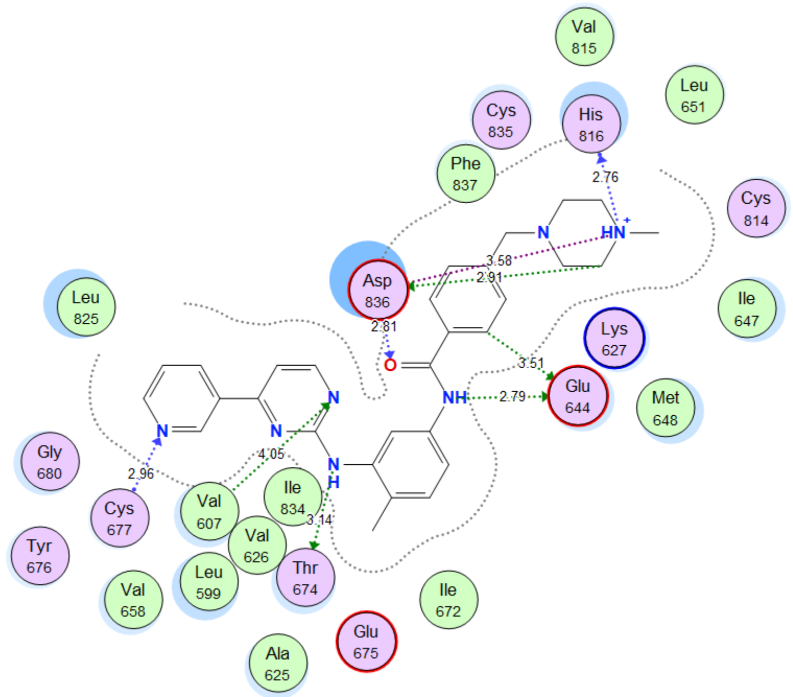

Figure 5: 2D interactions of **imatinib** within **PDGFRA** active site

Docking setup was first validated by self-docking of the co-crystallized ligand (**imatinib**) in the vicinity of the binding site of the enzyme, the docking score (S) was -18.0520 kcal/mol. and root mean square deviation (RMSD) was 0.6983 Å (**Figure 6**).

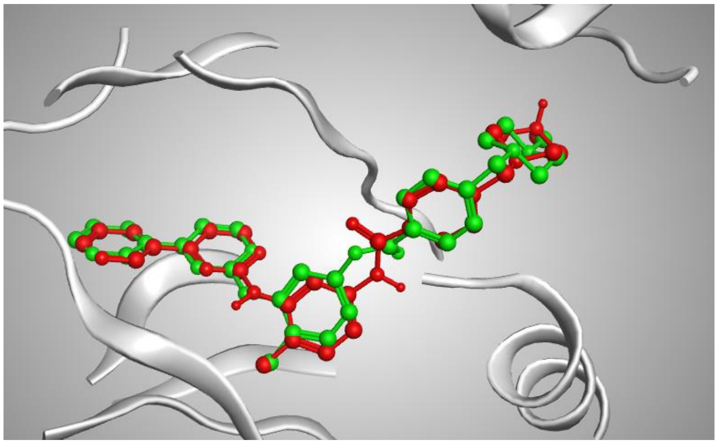

Figure 6: 3D representation of the superimposition of the co-crystallized (red) and the docking pose (green) of **imatinib** in the active site of **PDGFRA**

The **4j** compound showed similar energy binding score to that of **Sorafenib**. It exhibited good binding interactions with the amino acid in **PDGFRA** active site. The results are summarized in **Table (2)** & **Figures (7 & 8).**

Table (2): Docking results

Compound	S (kcal/mol)	Amino acids	Interacting groups	Type of interaction	Length
**4j**	-13.2171	Lys627	O (S=O)	H-bond acceptor	3.62
		Ile647	NH_2	H-bond acceptor	3.99
		Cys814	NH_2	H-bond donor	3.20
		Cys814	O (C=O)	Electrostatic	3.64
		His816	O (C=O)	H-bond acceptor	3.67
		Asp836	CH (Phenyl)	Electrostatic	2.83
		Asp836	CH	Electrostatic	3.42
**Sorafenib**	-13.0476	Glu644	NH	H-bond donor	2.87
		Glu644	NH	H-bond donor	3.26
		Met648	NH	H-bond acceptor	4.05
		Cys677	O (C=O)	H-bond acceptor	3.00
		Cys814	Cl	Halogen bond	3.97
		Asp836	O (C=O)	H-bond acceptor	2.88

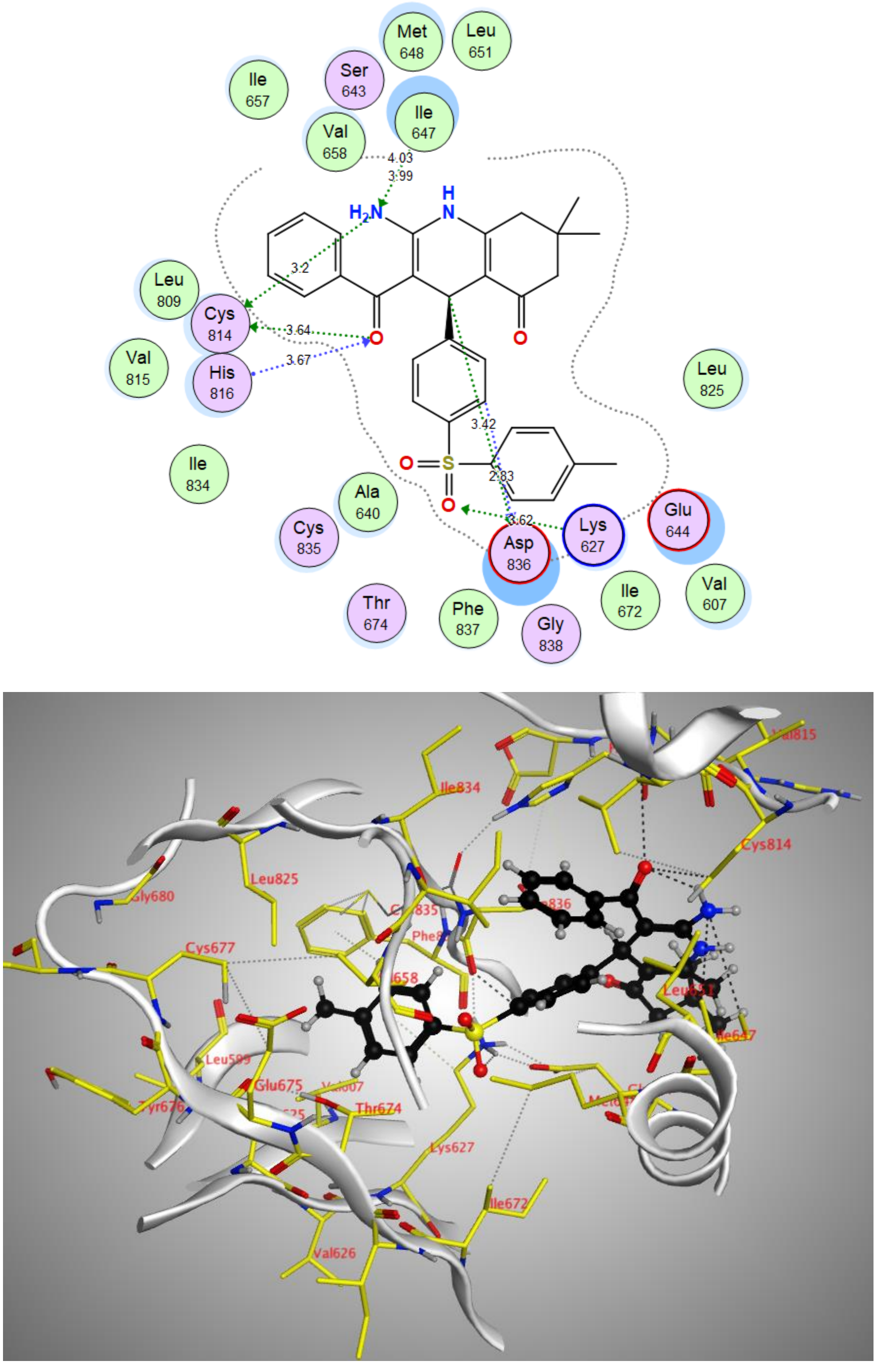

Figure 7: 2D & 3D diagram of compound **4j** interactions withing **PDGFRA** binding site

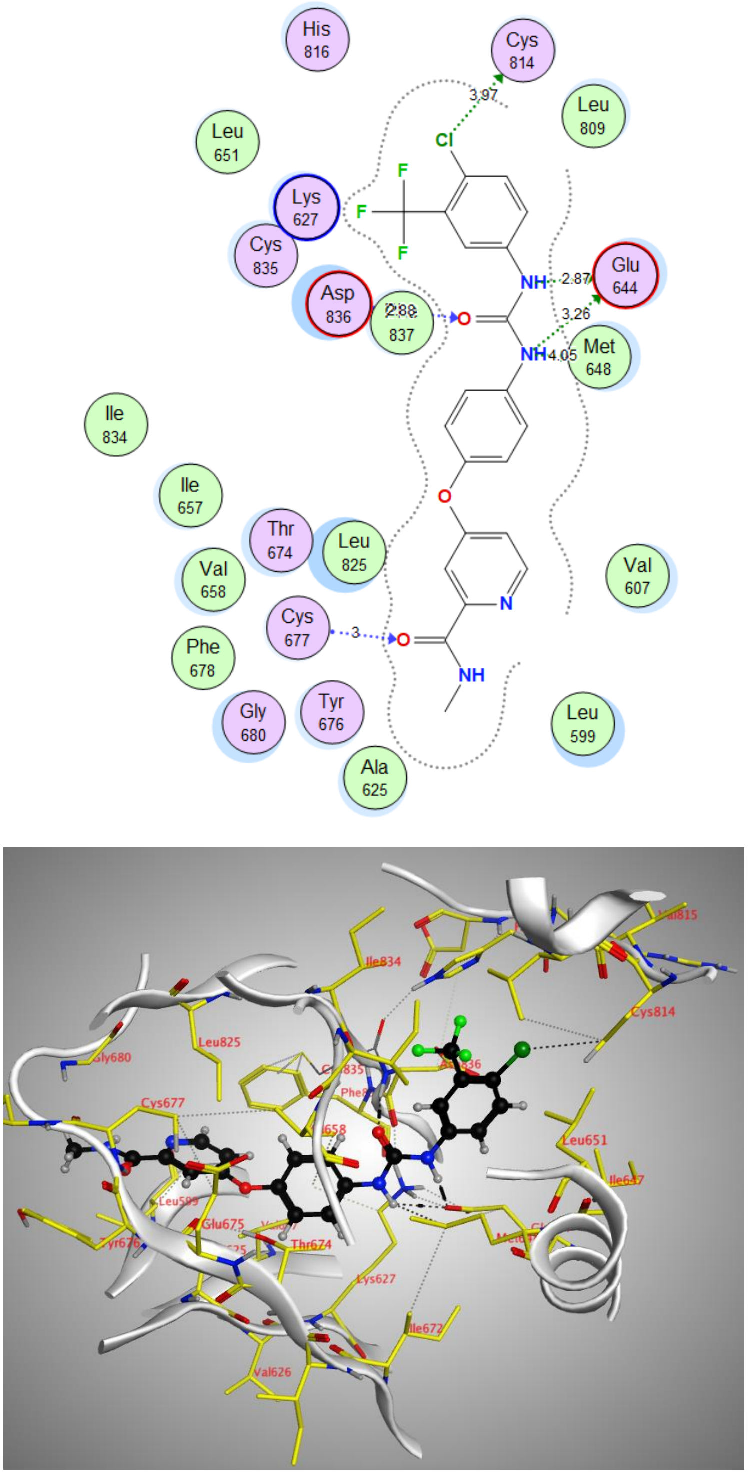

Figure 8: 2D & 3D diagram of **Sorafenib** interactions withing **PDGFRA** binding site

S3: NMR Data

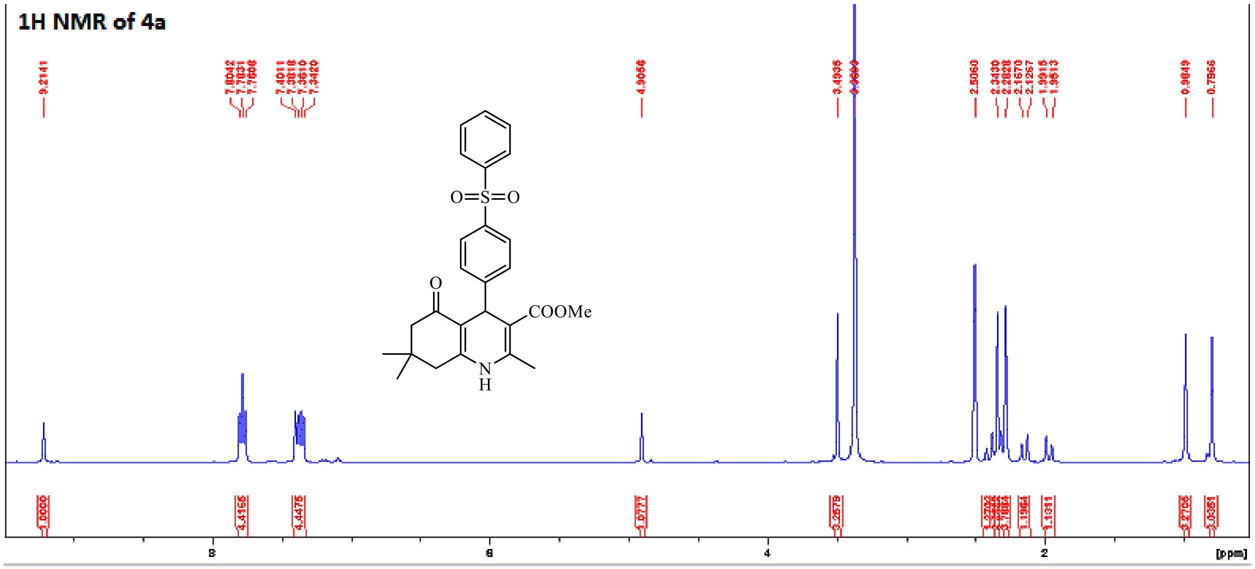
1H NMR of 4a
O=S=O
O
COOMe
N
H
8
6
4
2
[ppm]

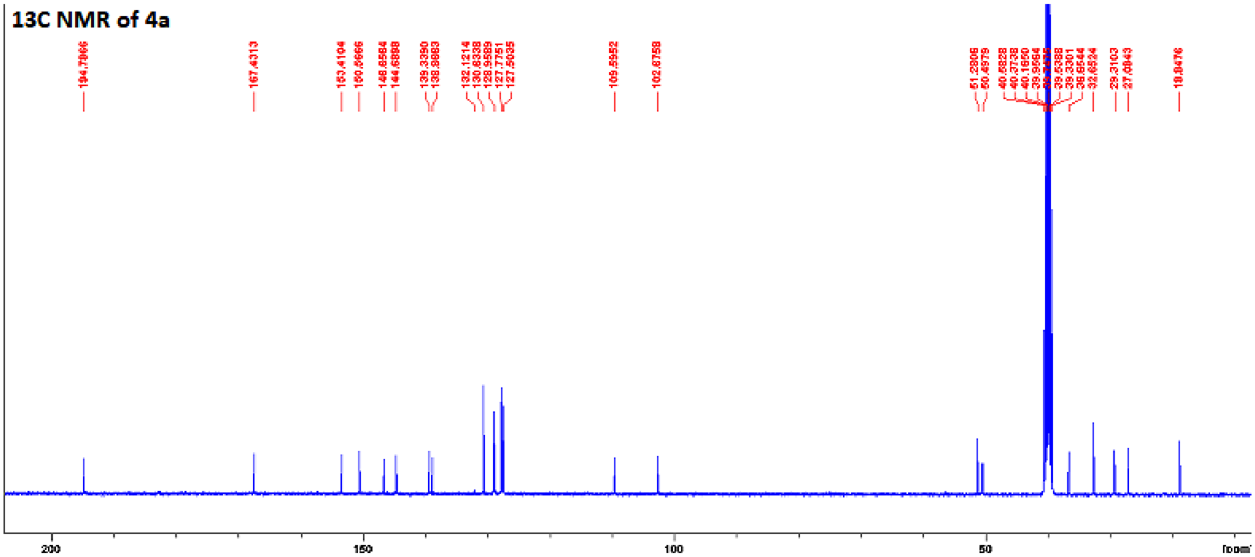
13C NMR of 4a
200
150
100
50
[ppm]

1H NMR 4b

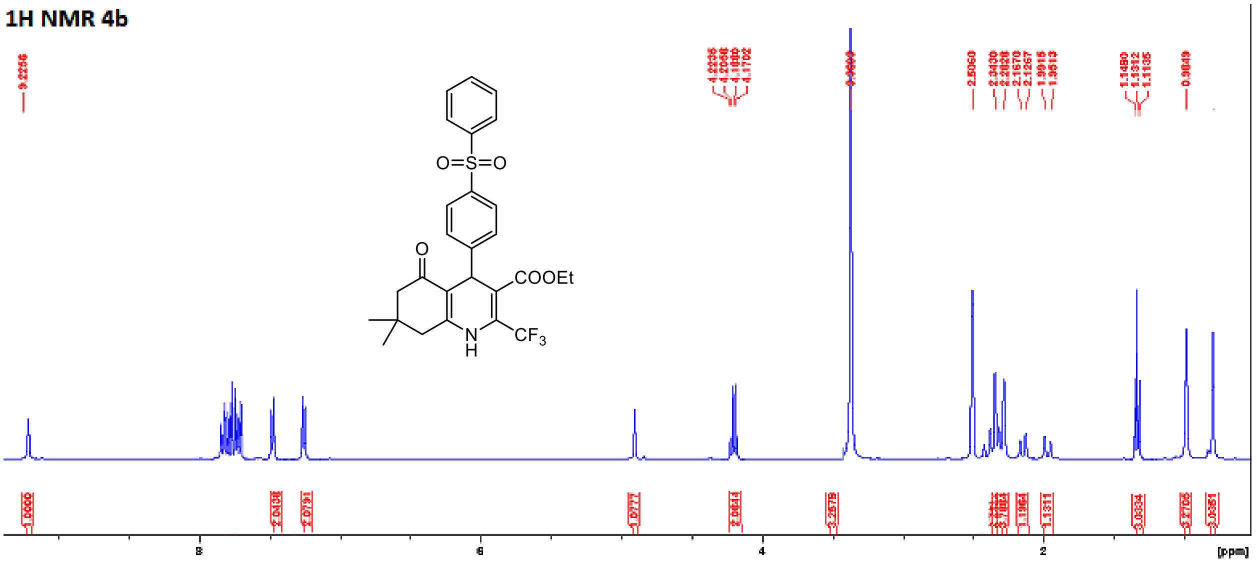

^{13}C NMR of 4b

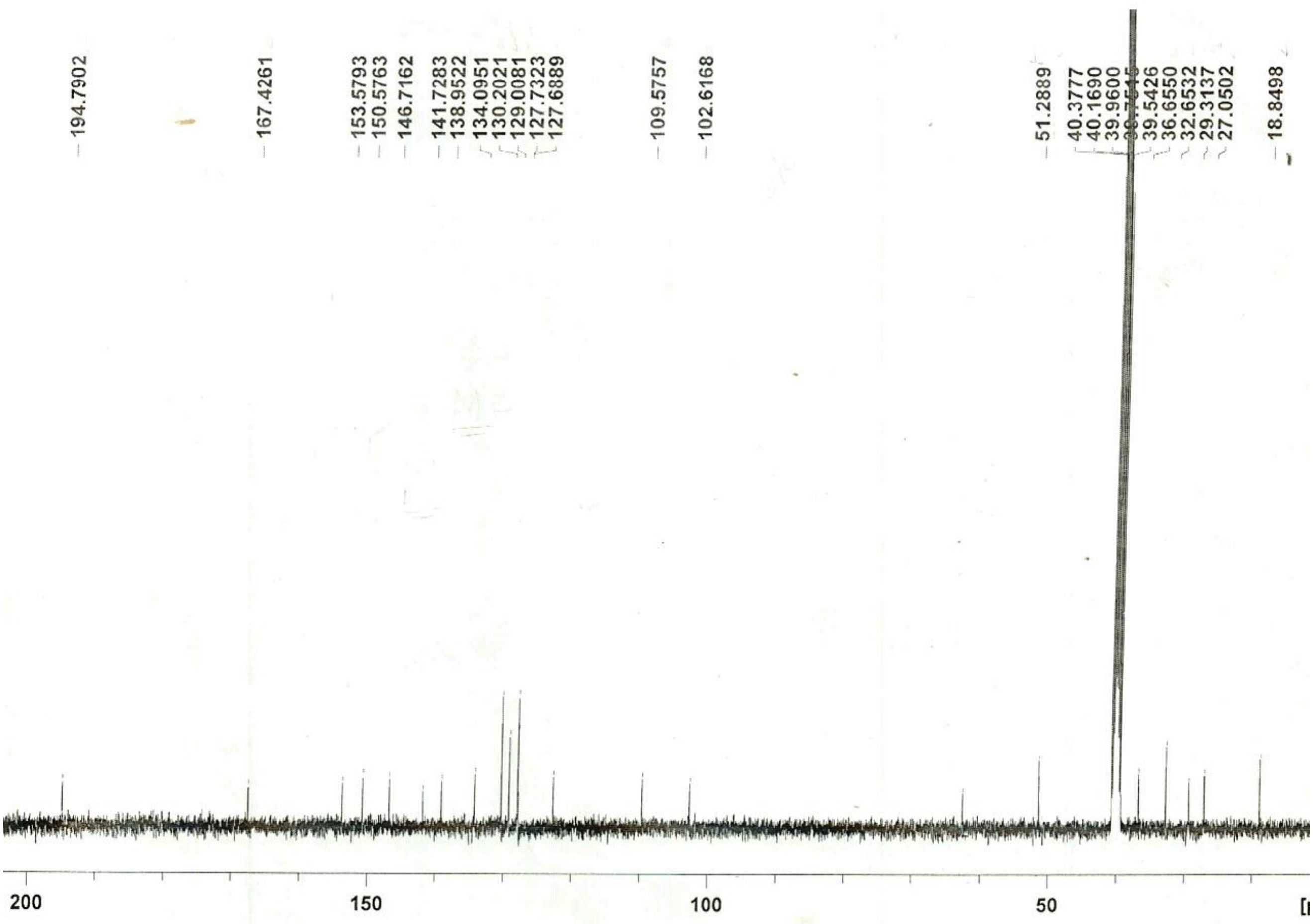

^{1}H NMR of Compound 4c

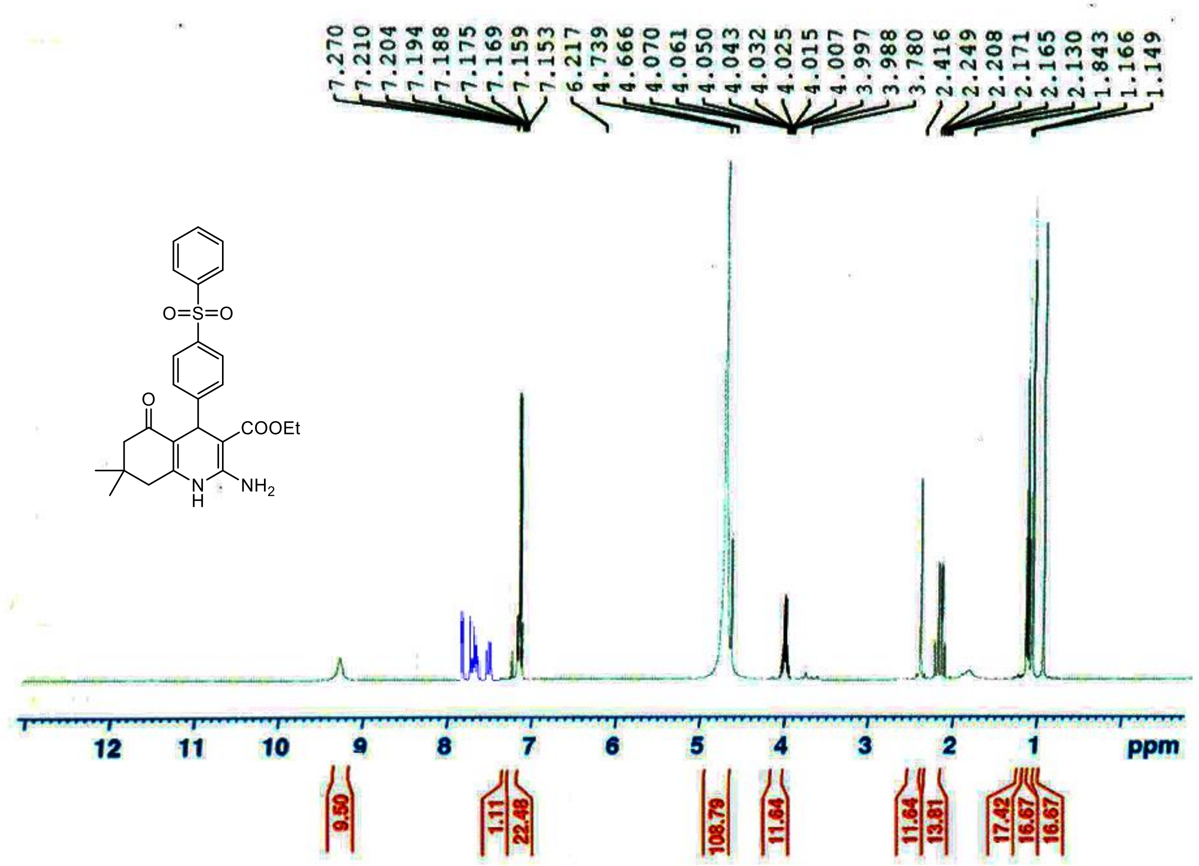

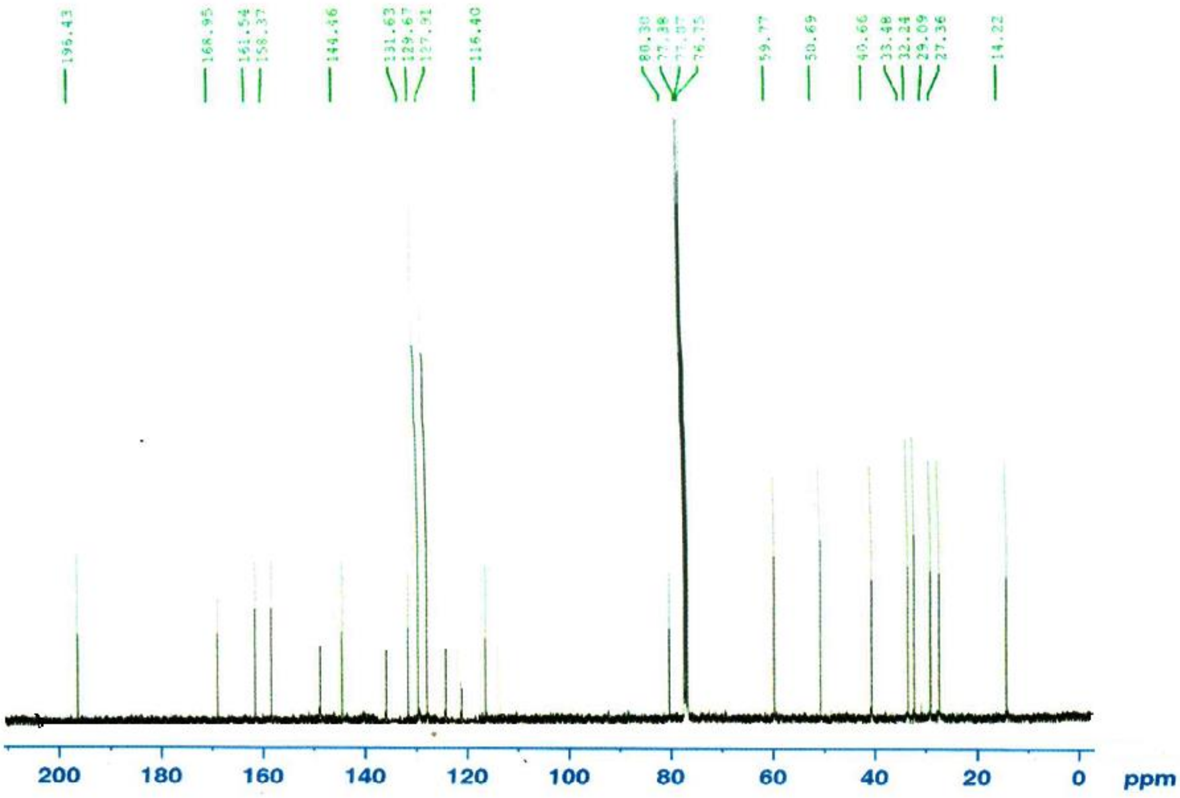
196.43
168.95
161.54
158.37
144.46
131.63
129.67
127.91
116.40
80.30
77.38
77.07
76.75
59.77
50.69
40.66
33.48
32.24
29.09
27.36
14.22
200
180
160
140
120
100
80
60
40
20
0
ppm

1H NMR of 4d

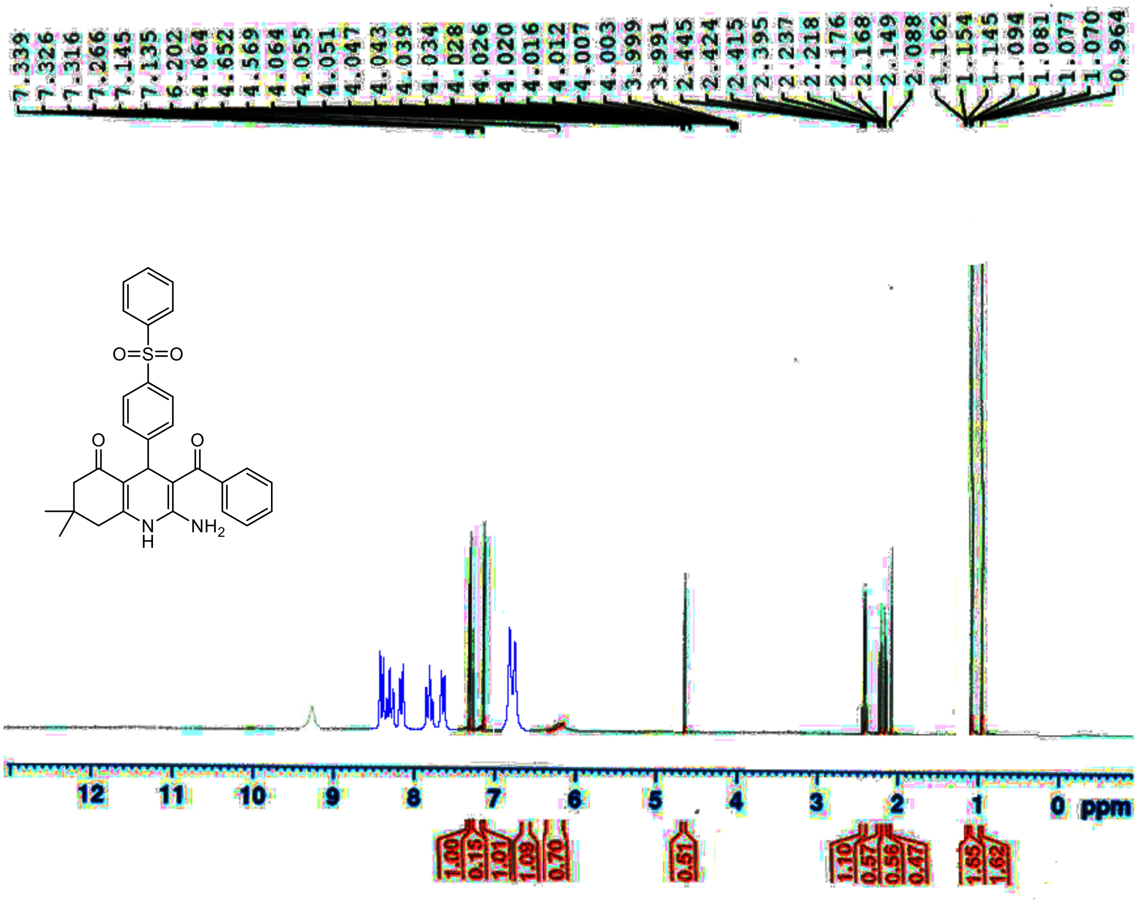

^{13}C NMR of 4d

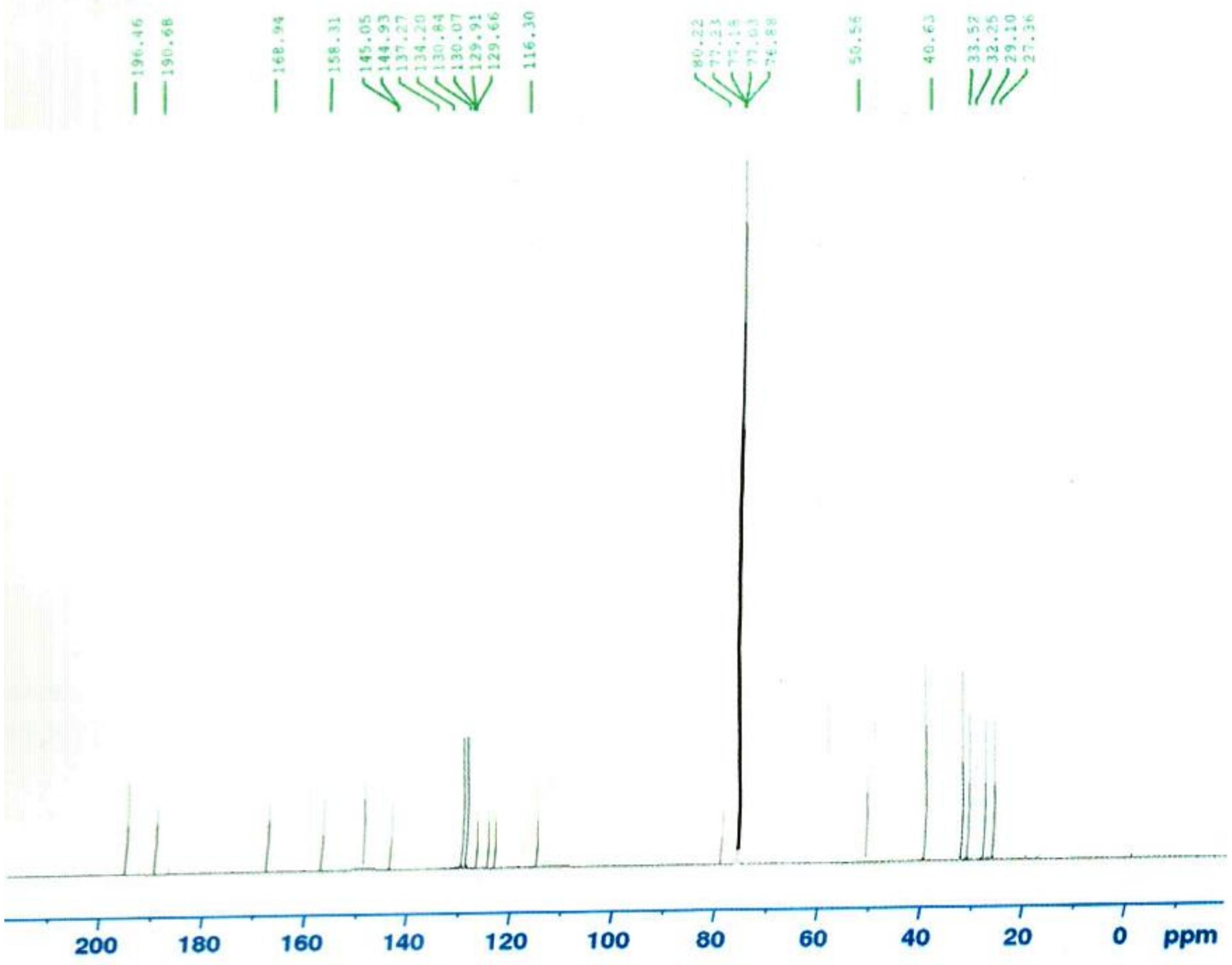
196.46
190.68
168.94
158.31
145.05
144.93
137.27
134.20
130.84
130.07
129.91
129.66
116.30
50.56
40.63
33.52
32.25
29.10
27.36
200
180
160
140
120
100
80
60
40
20
0
ppm

^{1}H NMR of compound 4e

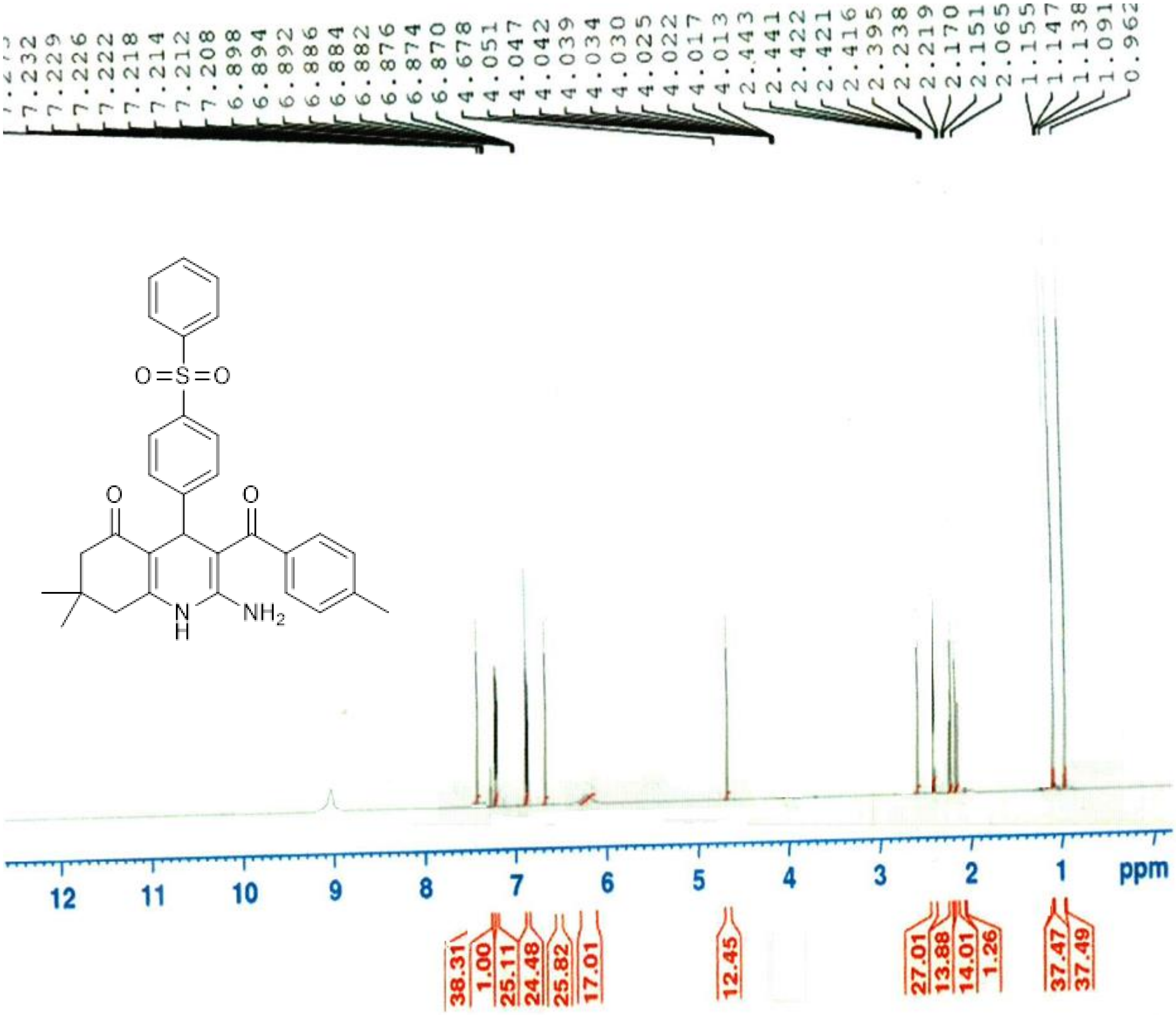

^{13}C NMR of 4e

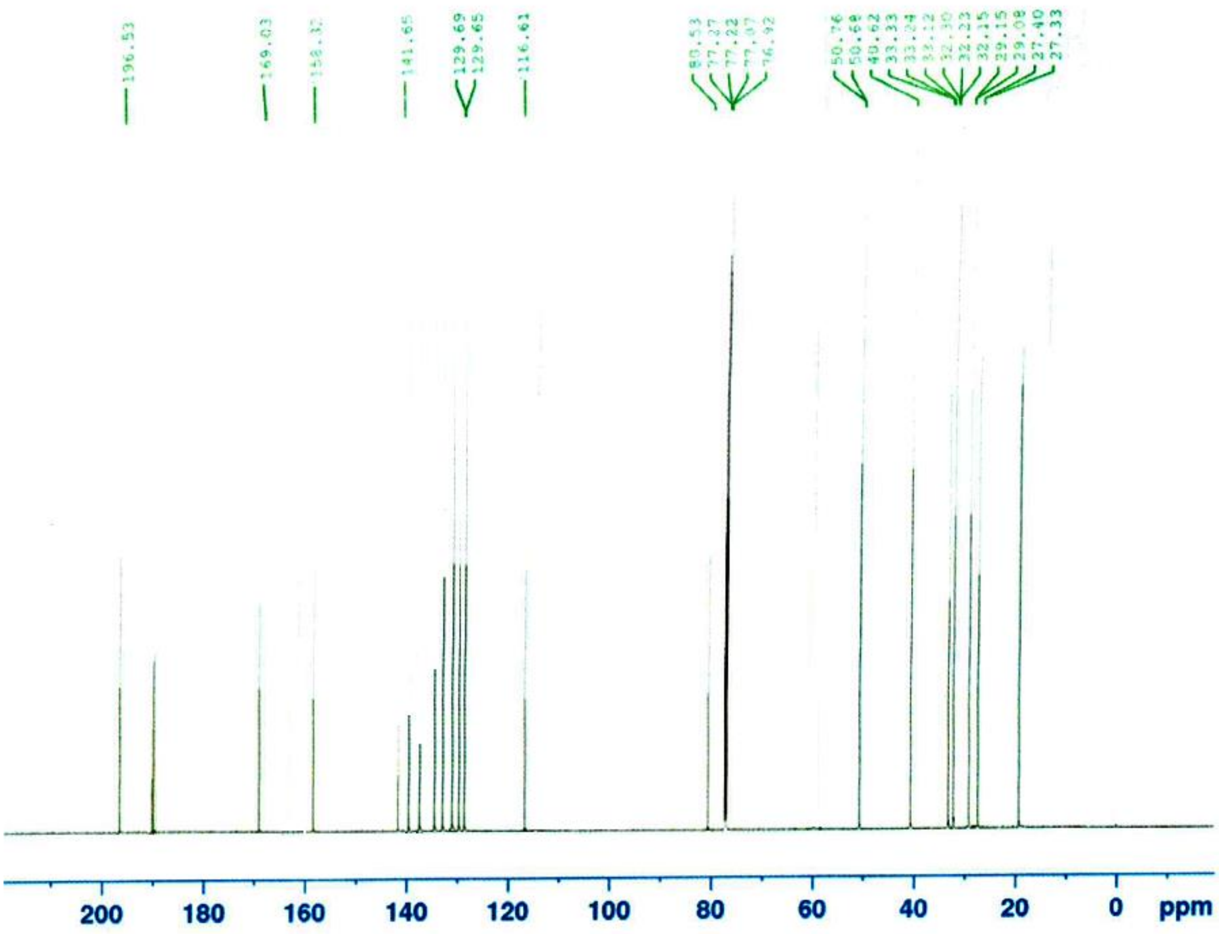

1H NMR of 4g

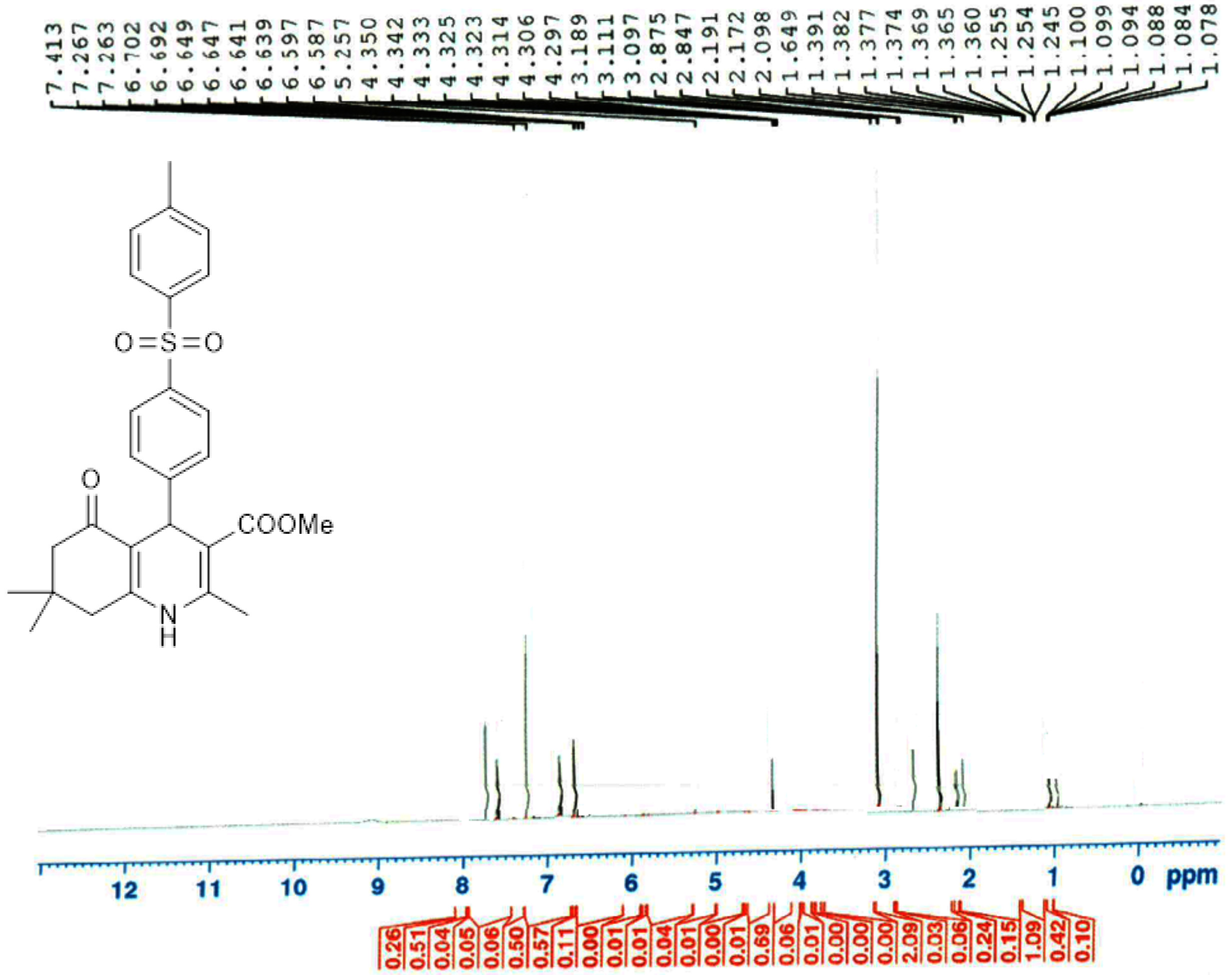

^{13}C NMR of 4g

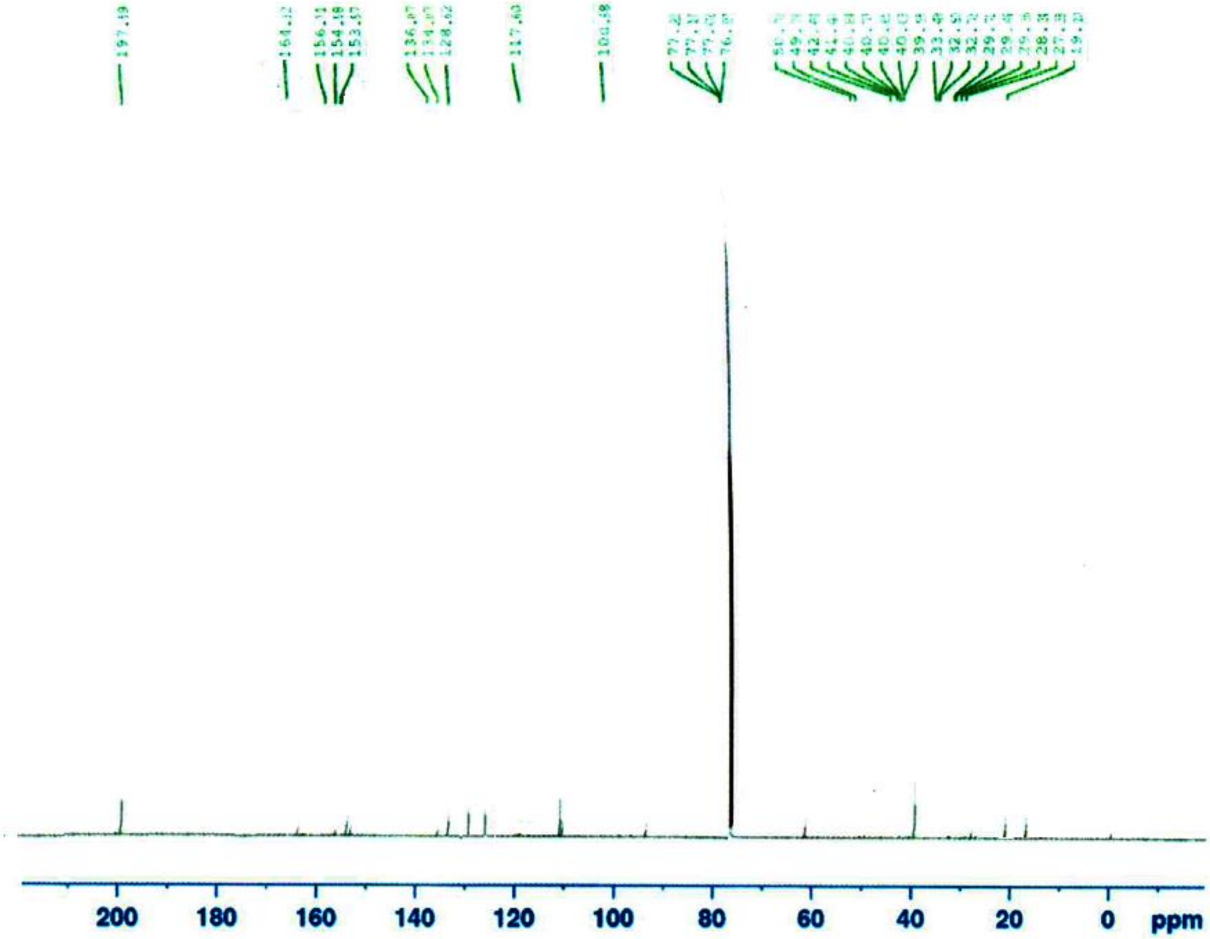

Supplement: Supplemental Material [file IENZ_A_1944126_SM8658.pdf]
